# Combustion Kinetics of Natural Polymers in Lemon-Tree Pruning Wood at High Heating Rates: Criado Master Plots and Šesták–Berggren Modelling

**DOI:** 10.3390/polym18192344

**Published:** 2026-09-25

**Authors:** Mohamed Anwar Ismail, Ibrahim Dubdub, Suleiman Mousa, Mohammed Al-Yaari

**Affiliations:** 1 Mechanical Engineering Department, College of Engineering, King Faisal University, P.O. Box 380, Al-Ahsa 31982, Saudi Arabia; maismail@kfu.edu.sa; 2 Chemical Engineering Department, College of Engineering, King Faisal University, P.O. Box 380, Al-Ahsa 31982, Saudi Arabia; saamousa@kfu.edu.sa (S.M.); malyaari@kfu.edu.sa (M.A.-Y.)

**Keywords:** lemon tree branches, lignocellulosic polymers, citrus pruning, biomass combustion, TGA kinetics, Criado master plot, Šesták–Berggren, solid biofuel

## Abstract

Lemon-tree pruning wood is a lignocellulosic natural-polymer solid whose cell wall is built from cellulose, hemicellulose and lignin. This study evaluates the use of this polymer matrix as a solid biofuel by combining physicochemical characterization with non-isothermal oxidative conversion at 20–80 K min^−1^. Lemon-tree branch (LTB) from orchards in Nubaria, Egypt, was analyzed for proximate and ultimate composition, Van Soest fiber fractions, HHV, FTIR, XRD and SEM–EDX. Thermogravimetric combustion was carried out in air at 20, 40, 60 and 80 K min^−1^. Apparent activation energies were obtained by five isoconversional methods (FR, FWO, KAS, STK, VY) and by Kissinger analysis of the two dry-fuel DTG peaks. Reaction models were screened by Criado master plots, a single-event Šesták–Berggren fit and Coats–Redfern. LTB is a moderately crystalline xylem (cellulose 52.11 wt%, CrI 52.6%, lignin 17.11 wt%) with moisture 7.10 wt%, volatile matter 77.80 wt%, fixed carbon 19.90 wt%, ash 2.30 wt% and HHV 18.50 MJ kg^−1^. Nitrogen and sulfur are 0.55 and 0.18 wt%. Combustion proceeds in three stages: Stage 1 moisture release, Stage 2 holocellulose-dominated oxidative devolatilization (65–68 wt% loss, T_p1_ ≈ 600–640 K) and Stage 3 char oxidation (≈25 wt% loss, T_p2_ ≈ 710–730 K). Mean isoconversional E_a_ on α = 0.1–0.7 is 98–115 kJ mol^−1^ and rises to ~212 kJ mol^−1^ at α = 0.8. Criado plots follow F2/F3/D3 up to α ≈ 0.5; a Šesták–Berggren function with *m* = 0.174 and *n* = 2.3 describes the Stage 2 window (R^2^ = 0.890) as an empirical fit only. Coats–Redfern selects F2 for Stage 2. ΔH tracks $E_{a}$, ΔG is positive, and ΔS is negative on Stage 2. LTB is a low-ash woody pruning fuel suitable for pellets or co-firing if Stage 3 is completed above ~750 K.

## 1. Introduction

The escalating global demand for energy, together with the depletion of fossil-fuel reserves and the associated burden of greenhouse-gas emissions, has intensified the search for renewable solid fuels [1]. Biomass from agricultural residues, forestry wastes and orchard prunings is an attractive candidate because it is widely available, notionally carbon-neutral and already handled as a waste stream [2]. Among the thermochemical options, direct combustion remains the most established route for heat and power, and it offers a practical alternative to open-field burning of pruning wood [3,4].

Citrus is one of the world’s major fruit crops, and commercial orchards generate a recurring woody residue that is rarely treated as a designed fuel. Velázquez-Martí et al. [5] reported an average of 2.68 t of dry pruning biomass per hectare for several orange cultivars and approximately 6.9 t ha^−1^ for high-yielding mandarin groups, with a mean gross calorific value of 17.4 MJ kg^−1^ for orange and mandarin wood. Formation pruning of young trees returns less wood than the fruiting-wood cuts applied to trees in full production [6]. Egypt is a relevant setting for this residue. National orange plantings are about 170,000 ha, or roughly 80% of Egypt’s citrus area [7], and the Nubaria district of Beheira Governorate is a principal production belt and a documented location of commercial lemon orchards [8]. Seasonal pruning in that belt produces lignified branch wood that is, at present, burned in the field or left to decay.

Published fuel data on lemon and citrus pruning support its use as a solid biofuel. Dağtekin et al. [9] pelletized lemon-orchard residues and obtained a gross calorific value of 18.60 MJ kg^−1^, 5.20 wt% ash, a bulk density of 521.33 kg m^−3^, a particle density of 1236.70 kg m^−3^ and a mechanical durability of 88.57%. García et al. [10] reported Spanish lemon-tree branches as 76.7 wt% volatile matter, 18.6 wt% fixed carbon, 4.7 wt% ash and 17.56 MJ kg^−1^. Palma et al. [11] reported citrus pruning as 48.86 wt% C, 5.75 wt% H, 0.35 wt% N and 1.80 wt% ash on a dry basis, with a lower heating value of 17.95 MJ kg^−1^ at 12.31% moisture. These values place citrus pruning among woody agricultural fuels, rather than high-ash herbaceous straws.

Reactor-scale studies confirm that ranking, and identify the operating constraints. Vamvuka et al. [3] combusted orange-tree pruning, leaves and peels in a laboratory fluidized bed, and found that raising the excess-air ratio or the feed rate improved burnout; carbon monoxide increased with volatile-matter and ash contents, sulfur dioxide was negligible, nitrogen oxides were generally high, and orange pruning gave the highest efficiency of the three streams. In an 80 kW boiler, citrus pellets carried more ash and moisture than olive-pellet mixtures, and blends containing citrus generated more CO and SO_2_ than olive residues alone [4]. Homogeneous citrus pruning also produced higher total polycyclic aromatic hydrocarbon emissions than olive or grapevine pruning, although the measured concentrations remained below the limits quoted in that study when the appliance was controlled [11]. These investigations establish woody citrus residues as credible boiler fuels. They do not provide conversion-resolved kinetic parameters for lemon-tree branch (LTB).

Thermogravimetric analysis (TGA) is the standard method for obtaining those parameters, and woody-pruning studies already show that combustion is multi-stage. Apple-pruning oxidation under oxygen exhibits distinct mass-loss regions for drying, holocellulose, lignin and char [12]. Olive-tree pruning under air follows drying, devolatilization and char oxidation, with a higher apparent activation energy than olive pomace [13], and fractionated olive wood confirms that lignin is the last constituent to oxidize [14]. For orange-tree pruning, Flynn–Wall–Ozawa activation energies under oxidative conditions at 5–20 K min^−1^ increase from about 78 kJ mol^−1^ at low conversion to about 247 kJ mol^−1^ at high conversion [15]. Almost all these datasets were acquired at laboratory heating rates of 5–20 K min^−1^. Industrial grate and fluidized-bed combustors impose steeper particle heating than the 5–20 K min^−1^ window used in most citrus-pruning TGA studies [2]. Programmed TGA rates of 20–80 K min^−1^ are closer to that practice, but they are not a one-to-one map of an industrial thermal gradient. Apparent kinetic parameters for that ramp window are, therefore, still required before the dataset is used in device calculations.

A second gap is the distinction between citrus peel and citrus wood. Ismail et al. [16] examined lemon peel at 20–80 K min^−1^ and reported an HHV of 23.02 MJ kg^−1^, 73.2 wt% volatile matter, four combustion stages and an apparent activation energy that reached approximately 304 kJ mol^−1^, with a potassium-rich ash. Peanut shell, a lignocellulosic hard residue examined at the same heating rates, provides a closer analogy to wood than peel does, but it is not citrus xylem [17]. Lemon peel is parenchymatous and pectin-rich; lemon-tree branch is lignified secondary wood. Combustion stages, slagging propensity and the conversion dependence of the activation energy cannot be assumed to transfer from one tissue to the other.

A third gap concerns mechanism. Many high-rate biomass studies evaluate the apparent activation energy by isoconversional methods and then assign a single solid-state model across the full conversion range by Coats–Redfern fitting. ICTAC recommendations require that $E_{a}$ first be obtained as a function of conversion without assuming a model, and that the model be identified by an independent diagnostic when $E_{a}$ is not constant [18,19]. Master-plot comparison of experimental reduced-rate data with theoretical solid-state curves [20,21] and empirical Šesták–Berggren [22,23] provide that diagnostic. Neither has been applied to LTB combustion at 20–80 K min^−1^. Lemon-tree branch is lignified secondary xylem whose cell wall is a natural-polymer composite of cellulose, hemicellulose and lignin. The present study addresses the gaps described above for that polymer matrix in pruning branches (*Citrus limon*) collected in the Nubaria basin. The objectives are (1) to determine the proximate and ultimate composition, fiber fractions, higher heating value and structural features of LTB by FTIR, XRD and SEM–EDX; (2) to resolve its oxidative decomposition at 20, 40, 60 and 80 K min^−1^ and to evaluate ignition, burnout and the combustibility index; (3) to obtain the conversion-dependent apparent activation energy using five isoconversional methods (Friedman, Flynn–Wall–Ozawa, Kissinger–Akahira–Sunose, Starink, and Vyazovkin) and Kissinger on the two reactive peaks, and to screen candidate solid-state models by the Coats–Redfern method; and (4) to identify the rate-limiting mechanism using Criado master plots and Šesták–Berggren modelling, and to calculate the associated thermodynamic parameters. The resulting dataset is intended to support the evaluation of LTB as a solid biofuel and the design of combustion systems for citrus/pruning wood.

## 2. Materials and Methods

### 2.1. Biomass Sourcing and Conditioning

Pruning wood of mature lemon trees (*Citrus limon*) was collected after routine orchard cuts in the Nubaria agricultural district, Beheira Governorate, Egypt. Leafy twigs and fruit were discarded so that the working sample represented lignified branch wood. The branches were rinsed with deionized water to remove adhering soil, and oven-dried at 310 K for 24 h in a Memmert UN110 oven (Memmert GmbH + Co. KG, Schwabach, Germany). Soluble alkali salts may have been reduced by this wash, relative to unwashed orchard wood; inorganic results below are therefore reported for the washed fuel. Dried wood was milled in an IKA MF 10 Basic mill (IKA-Werke GmbH & Co. KG, Staufen, Germany) and classified on an ELE Sieve-Tronic shaker (ELE International, Milton Keynes, UK). The mass-weighted mean particle size was 0.306 mm. The powder was stored in sealed glass jars at 298 K. Proximate, ultimate, fiber and TGA values are technical triplicates of this homogenized batch, not independent biological replicates from separately sampled trees.

### 2.2. Fuel Characterization

The instruments, standards, and operating conditions used for proximate, ultimate, calorimetric, and fiber analyses are summarized in Table 1. All compositional tests were performed in triplicate.

Fixed carbon and oxygen were obtained by difference:

(1) $$FC\;\left(wt\%\right)=100-\left(VM+Ash\right)\;O\;\left(wt\%\right)=100-\left(C+H+N+S+Ash\right)$$

Hemicellulose, cellulose, and extractives were calculated from the Van Soest residuals [27]:

(2) Hemicellulose=NDF−ADFCellulose=ADF−ADLExtractives=100−NDF

LTB is lignified secondary wood, rather than pectin-rich peel, so the Van Soest fractions are interpreted as structural hemicellulose, cellulose, and lignin.

### 2.3. Spectroscopic and Microstructural Analysis

#### 2.3.1. Fourier-Transform Infrared Spectroscopy

Transmission spectra were recorded on a JASCO FTIR-4100 spectrometer (JASCO Corporation, Tokyo, Japan) from KBr discs (2 mg LTB + 200 mg KBr) over 4000–400 cm^−1^ at 4 cm^−1^ resolution (32 scans).

#### 2.3.2. Powder X-Ray Diffraction

Diffraction patterns were collected on a Rigaku Ultima IV diffractometer (Rigaku Corporation, Akishima, Tokyo, Japan) with Cu Kα radiation (λ=0.15406 nm) at 40 kV and 30 mA, scanning $2\theta =10^{{}^{\circ}}-90^{{}^{\circ}}$ at 1° min^−1^ with a 0.02° step. The Segal crystallinity index [28] was

(3) $$CrI\;\left(\%\right)=\frac{I_{002}-I_{am}}{I_{002}}\times 100$$

where $I_{002}$ is the Cellulose I intensity near $2\theta \approx 22^{{}^{\circ}}$ and $I_{am}$ is the amorphous intensity near $2\theta \approx 18^{{}^{\circ}}$.

#### 2.3.3. Scanning Electron Microscopy and Energy-Dispersive X-Ray Spectroscopy

Particle morphology was examined on a TESCAN VEGA3 scanning electron microscope (TESCAN, Brno, Czech Republic) equipped with an integrated secondary-electron detector, operated at 20.0 kV with a working distance of 4.9 mm. Energy-dispersive spectra and maps were acquired on the same instrument with a Bruker XFlash 5010 detector and Esprit software (Bruker Nano, Berlin, Germany; software version: ESPRIT 2.1).

### 2.4. Non-Isothermal Combustion Experiments

Oxidative mass-loss profiles were recorded on a NETZSCH STA 449 F3 Jupiter analyzer (NETZSCH-Gerätebau GmbH, Selb, Germany) following ICTAC practice for non-isothermal kinetic experiments [18]. Approximately 4.2 mg (4.16–4.33 mg) of LTB was loaded, without compaction, into an alumina crucible and heated from 298 K to 1273 K (25–1000 °C) under synthetic air (20% $O_{2}$/80% $N_{2}$) flowing at 60 mL min^−1^. Four linear heating rates were used: $\beta =20,\;40,\;60,\;\mathrm{and}\;80{\;K\;\min}^{-1}$. TG and DTG traces were recorded in triplicate; reported curves are averages. Replicate mass-loss and peak-temperature deviations were within ±1.5% and ±2 K, respectively.

The conversion was defined as

(4) $$\alpha =\frac{m_{0}-m_{T}}{m_{0}-m_{\infty }}$$

where $m_{0}$, $m_{T}$, and $m_{\infty }$ are the initial mass, the mass at temperature T, and the residual mass, respectively. Ignition ($T_{i}$) and burnout ($T_{b}$) temperatures were determined from the TG–DTG records via the intersection method. The combustibility index was calculated as

(5) $$S=\frac{{\left(d\alpha /dt\right)}_{max}\cdot {\left(d\alpha /dt\right)}_{mean}}{T_{i}^{2}\cdot T_{b}}$$

with peak and mean conversion rates taken between $T_{i}$ and $T_{b}$, and temperatures expressed in kelvin. Characteristic DTG maxima used later as *T*_p1_ (Stage 2, oxidative devolatilization) and *T*_p2_ (Stage 3, char oxidation) were taken from the dry-fuel peaks above 450 K. The weak Stage 1 moisture maximum was not used in the Kissinger or thermodynamic calculations. Programmed rates of 20–80 K min^−1^ were chosen because they lie closer to particle heating rates for grate and fluidized-bed practice than the 5–20 K min^−1^ window used in most citrus-pruning TGA studies. They are not a one-to-one map of an industrial thermal gradient; the kinetic parameters are apparent values for 0.306 mm particles under these ramps.

### 2.5. Kinetic and Mechanistic Analysis

Under a linear temperature ramp, the heterogeneous reaction rate is described by

(6) $$\frac{d\alpha }{dT}=\frac{A}{\beta }\exp\left(-\frac{E_{a}}{RT}\right)f\left(\alpha \right)$$

where A is the pre-exponential factor ($\min^{-1}$), $E_{a}$ is the apparent activation energy (${\mathrm{kJ}\;\mathrm{mol}}^{-1}$), $R=8.314{\;J\;\mathrm{mol}}^{-1}{\;K}^{-1}$, and $f\left(\alpha \right)$ is the differential reaction model.

#### 2.5.1. Isoconversional Activation Energies

Apparent $E_{a}\left(\alpha \right)$ was evaluated at α=0.10–0.90 (Δα=0.10) by five isoconversional methods: Friedman (FR) (differential); Flynn–Wall–Ozawa (FWO), Kissinger–Akahira–Sunose (KAS), Starink (STK) (integral approximations); and the non-linear Vyazovkin (VY) integral [18,19]. Working equations and regression variables are summarized in Table S1 (Supplementary Materials).

#### 2.5.2. Coats–Redfern Method

Stage-wise windows identified on the DTG curves were fitted using the Coats–Redfern (CR) integral equation [29]:

(7) $$\ln\left[\frac{g\left(\alpha \right)}{T^{2}}\right]=\ln\left(\frac{AR}{\beta E_{a}}\right)-\frac{E_{a}}{RT}$$

against sixteen standard solid-state models spanning reaction order (F1–F3), diffusion (D1–D4), nucleation (A2–A4), geometrical contraction (R1–R3), and power law (P2–P4). The corresponding $g\left(\alpha \right)$ functions are listed in Table S2 (Supplementary Materials). The linearization was applied separately to Stage 2 (oxidative devolatilization) and Stage 3 (char oxidation). Moisture is only a small fraction of the full-run conversion, so Stage 2 begins after drying. The moisture interval was not fitted. Coats–Redfern rankings were used to identify candidate mechanisms on Stage 2. The Stage 3 triplet was screened, and is reported only where the regression is physically defined (*E*_a_ > 0); it is not used for design.

#### 2.5.3. Criado Master Plots

Experimental reduced-rate curves were compared with theoretical $z\left(\alpha \right)$ master curves [20,21]. The normalized working equation independent of initial $g\left(\alpha \right)$ assumptions is

(8) $$\frac{z\left(\alpha \right)}{z\left(0.5\right)}=\frac{{\left(d\alpha /dT\right)}_{\alpha }}{{\left(d\alpha /dT\right)}_{0.5}}{\left(\frac{T_{\alpha }}{T_{0.5}}\right)}^{2}=\frac{f\left(\alpha \right)g\left(\alpha \right)}{f\left(0.5\right)g\left(0.5\right)}$$

Experimental $z\left(\alpha \right)/z\left(0.5\right)$ profiles were calculated at each heating rate and overlaid onto theoretical curves from Table S2 to diagnose controlling mechanisms across conversion intervals.

#### 2.5.4. Šesták–Berggren Model

Where the master plot departed from idealized models, the empirical single-event Šesták–Berggren function [22,23] was fitted to the conversion data:

(9) $$f\left(\alpha \right)=c\cdot \alpha^{m}{\left(1-\alpha \right)}^{n}$$

The kinetic exponents m and n, along with the scaling constant c, were resolved through non-linear regression by minimizing the sum of squared residuals (SSE) between experimental and calculated $z\left(\alpha \right)/z\left(0.5\right)$ profiles. The single-event (*m*, *n*) pair is an empirical description of Stage 2. It is not treated as a reactor-scale rate law, and is not applied to Stage 3.

### 2.6. Thermodynamic Parameters and Kinetic Compensation Effect

Enthalpy (ΔH), Gibbs free energy (ΔG), and entropy (ΔS) of activation were calculated from transition-state theory at the DTG peak temperature ($T_{p}$):

(10) $$\Delta H=E_{a}-RT_{p}$$

(11) $$\Delta G=E_{a}+RT_{p}\ln\left(\frac{k_{B}T_{p}}{hA_{0}}\right)$$

(12) $$\Delta S=\frac{\Delta H-\Delta G}{T_{p}}$$

where $k_{B}=1.381\times 10^{-23}{\;J\;K}^{-1}$ and $h=6.626\times 10^{-34}\;J\;s$. The pre-exponential factor ($A_{0}$) was evaluated using the mechanism validated by the Criado and Coats–Redfern analyses. The Kinetic Compensation Effect (KCE) was evaluated via linear regression of ${\ln A}_{0}$ against $E_{a}$ across conversion:

(13) $${\ln A}_{0}=a+bE_{a}$$

from which the isokinetic temperature ($T_{iso}$) was calculated as $T_{iso}=1/\left(bR\right)$.

## 3. Results and Discussion

### 3.1. Physicochemical Characterization

The proximate, ultimate, calorimetric and Van Soest composition of LTB is given in Table 2. Moisture is reported as received; all other figures are on a dry basis. Fixed carbon and oxygen were obtained by difference, as defined in Section 2.2. Values are means of triplicate determinations.

The as-received moisture (7.10 ± 0.15 wt%) lies in the range expected for oven-dried orchard wood and is below the 10 wt% threshold usually specified for small-boiler fuels [9]. Ash is 2.30 ± 0.10 wt%, comparable to citrus pruning (1.80 wt%; [11]) and Spanish lemon-tree branches (4.7 wt%; [10]), and well below lemon-orchard pellets (5.20 wt%; [9]) and lemon peel [16]. A low ash burden reduces the slagging and handling load in a grate or fluidized bed. Volatile matter is 77.80 ± 0.35 wt% and fixed carbon 19.90 ± 0.25 wt%. Both sit inside the citrus-wood band reported for Spanish lemon-tree branches (VM 76.7 wt%, FC 18.6 wt%, ash 4.7 wt%, HHV 17.56 MJ kg^−1^; [10]). The VM/FC ratio is 3.91, which indicates a fuel that ignites readily and releases most of its energy in the gas phase, with a smaller char-oxidation tail. Lemon peel examined at the same heating rates has a lower volatile fraction (73.2 wt%) and a higher HHV (23.02 MJ kg^−1^), reflecting a pectin- and extractive-rich tissue, rather than xylem [16].

Elemental carbon (48.30 ± 0.20 wt%) and hydrogen (5.90 ± 0.12 wt%) match citrus chips (C 48.86 wt%, H 5.75 wt%; Palma et al., 2023 [4]). The atomic H/C and O/C ratios are 1.46 and 0.67, which place LTB with woody biomass, rather than with pectin-rich peels. Nitrogen (0.55 ± 0.04 wt%) and Sulphur (0.18 ± 0.02 wt%) are low, and imply a limited NO_x_ and SO_2_ inventory relative to many herbaceous residues, consistent with the negligible SO_2_ reported for orange-pruning combustion in a fluidized bed [3]. Oxygen by difference is 42.77 ± 0.22 wt%. Van Soest fractionation confirms the woody assignment. Cellulose is the majority constituent (52.11 ± 0.35 wt%), with hemicellulose 15.42 ± 0.50 wt%, lignin 17.11 ± 0.11 wt% and extractives 15.36 ± 0.28 wt%. Combined holocellulose (67.53 wt%) accounts for the high volatile fraction: cellulose and hemicellulose depolymerize in the main DTG peak. Lignin (17.11 wt%) underpins the fixed-carbon yield (19.90 wt%), because the phenylpropanoid network resists low-temperature volatilization and forms the char that burns at higher temperature. A stoichiometric reconstruction from the carbon contents of cellulose (44.4 wt% C), xylan-type hemicellulose (45.5 wt% C), hardwood lignin (~63.5 wt% C) and woody extractives (~58 wt% C) predicts ~49.9 wt% organic carbon, or ~48.8 wt%, after the 2.30 wt% ash. The measured carbon (48.30 ± 0.20 wt%) agrees with that estimate.

The measured HHV is 18.50 ± 0.25 MJ kg^−1^ (4422 ± 60 cal g^−1^). That value lies next to lemon-orchard pellets (18.60 MJ kg^−1^; [9]) and above Spanish lemon-tree branches (17.56 MJ kg^−1^; [10]) and the mean gross calorific value of orange and mandarin wood (17.4 MJ kg^−1^; [5]). It is lower than lemon peel (23.02 MJ kg^−1^; [16]), as expected for a fuel with less extractive oil and more structural polysaccharide. Applying the correlation of Channiwala and Parikh [30] to the measured C, H, N, S, O and ash gives 19.35 MJ kg^−1^, within 5% of the bomb value. Taken together, Table 2 supports the use of LTB as a solid biofuel: low moisture and ash, a high volatile fraction for ignition, modest N and S, and an HHV in the established citrus-pruning range. The fiber composition also fixes the interpretation of the TGA stages that follow. Devolatilization should be dominated by hemicellulose and cellulose; the high-temperature tail should belong to lignin and char.

### 3.2. Spectroscopic and Microstructural Characterization

FTIR, XRD and SEM–EDX of LTB are shown in Figure 1 and Figure 2. The three techniques are read against the fiber and ash figures in Table 2.

#### 3.2.1. FTIR

The spectrum is that of a lignocellulosic solid, not of a pectin-rich peel. A broad O–H stretch at 3423 cm^−1^ records hydroxyl groups of cellulose, hemicellulose and adsorbed moisture (7.10 wt%, Table 2). The band at 2920 cm^−1^ is aliphatic C–H stretching from polysaccharide and extractive methylene groups. The carbonyl at 1737 cm^−1^ is assigned to acetyl and uronic C=O of hemicellulose and to unconjugated carbonyls in lignin and extractives. That assignment is consistent with 15.42 wt% hemicellulose and 15.36 wt% extractives. The band at 1628 cm^−1^ is the aromatic C=C stretch of lignin plus bound water; it is the spectroscopic counterpart of the 17.11 wt% Klason lignin. The 1247 cm^−1^ band is C–O stretching in lignin (guaiacyl/syringyl) and in hemicellulose. The strongest fingerprint absorption, 1047 cm^−1^, is the C–O–C stretch of the cellulose pyranose ring, and matches the 52.11 wt% cellulose. No intense pectin carboxylate envelope in the 1600–1400 cm^−1^ region is present. The infrared spectrum, therefore, supports the Van Soest assignment in Table 2: LTB is secondary xylem.

#### 3.2.2. XRD

The diffractogram is a broad cellulose-I pattern. The principal maximum lies at $2\theta =21.5^{{}^{\circ}}$, the (002) reflection of native cellulose, sitting on an amorphous halo that rises from about $15^{{}^{\circ}}$ to $19^{{}^{\circ}}$. No sharp inorganic reflections are resolved between $30^{{}^{\circ}}$ and $50^{{}^{\circ}}$, in agreement with the low ash (2.30 wt%). The (002) maximum occurs at $2\theta =21.5^{{}^{\circ}}$ ($I_{002}=190$ counts). The amorphous intensity at $2\theta =18.0^{{}^{\circ}}$ is $I_{am}=90$ counts. The Segal crystallinity index is therefore 52.6%, consistent with a cellulose-rich but only moderately ordered xylem, and with the 52.11 wt% cellulose in Table 2. No discrete mineral reflections are resolved, in agreement with the 2.30 wt% ash.

#### 3.2.3. SEM–EDX

At 4 k× (Figure 2a) the powder is a mixture of irregular angular fragments and occasional slender fibers, with a characteristic length of a few tens of micrometers. At 15 k× (Figure 2b), an isolated fiber or vessel element is resolved, with attached fines. At 45 k× (Figure 2c), the fiber surface is rough and carries sub-micrometer debris. The morphology is milled xylem, not the collapsed parenchyma of citrus peel.

The maps in Figure 2d–g show carbon and oxygen distributed over the whole particle, as required for a polysaccharide–lignin matrix. Calcium is sparse, and locally clustered (Figure 2g). The EDX quantification (Figure 2h) gives C 65.71 wt% (72.08 at%), O 33.65 wt% (27.71 at%) and Ca 0.64 wt% (0.21 at%). No K, Si, S or Cl peak is reported in the quantification table. The EDX carbon is higher than the bulk CHNS value (48.30 wt%) because EDX does not count hydrogen, is surface-weighted, and includes contribution from the carbon support. The C/O mass ratio from EDX is 1.95; the bulk C/O from Table 2 is 1.13. The difference is expected, and does not contradict the ultimate analysis. The only detected inorganic cation is calcium, which is the ash chemistry of wood (alkaline-earth), not of lemon peel (K-rich; [16]). A 0.64 wt% Ca surface signal on a 2.30 wt% ash fuel is consistent with a small Ca-bearing mineral fraction and with the absence of sharp XRD mineral peaks.

### 3.3. Combustion Behaviour

Non-isothermal combustion profiles of LTB at 20, 40, 60 and 80 K min^−1^ are shown in Figure 3. Characteristic temperatures, peak rates and stage mass losses are listed in Table 3. Ignition temperature and the combustibility index S are shown in Figure 4.

Three stages are resolved on every DTG curve (Figure 3a) and on the conversion traces (Figure 3b). The first stage, between approximately 300 and 420 K, is dehydration. The mass loss is 5 wt% at every heating rate and matches the as-received moisture of 7.10 ± 0.15 wt% in Table 2, once the bound-water tail into the onset of devolatilization is allowed for. The DTG peak in this window is weak (2–4 wt% min^−1^), and shifts from 350 K at 20 K min^−1^ to 375 K at 80 K min^−1^. The second stage is the main devolatilization. It begins near 500 K at 20 K min^−1^ and near 550 K at 80 K min^−1^, and ends near 650–690 K. The DTG maximum moves from 600 K to 640 K as the heating rate increases. Mass loss in this window is 65–68 wt%. That interval accounts for most of the 77.80 ± 0.35 wt% volatile matter in Table 2, and is consistent with the 67.53 wt% holocellulose (cellulose 52.11 wt% plus hemicellulose 15.42 wt%). Extractives (15.36 wt%) likely contribute to the low-temperature shoulder of the same peak. The DTG maximum is not resolved into named polymer sub-peaks at these heating rates. On the conversion plot the stage spans approximately α=0.05–0.70. The peak rate rises from 19 wt% min^−1^ at 20 K min^−1^ to 77 wt% min^−1^ at 80 K min^−1^ because the same volatile release is compressed into a shorter time. The third stage is char oxidation. It occupies 680–800 K, with a DTG maximum that moves from 710 K to 730 K. Mass loss is 25 wt% at every heating rate. That figure is consistent with the 19.90 ± 0.25 wt% fixed carbon plus the 17.11 ± 0.11 wt% lignin that remains after Stage 2. Stage 3 is assigned to lignin/char on that basis, not as proof that lignin burns in isolation. Residual mass after 900 K is about 2 wt%, and matches the 2.30 ± 0.10 wt% ash. Conversion in this window is approximately α=0.70–0.98. The high-temperature DTG peak is broader and lower than the holocellulose peak, as expected for a cross-linked phenylpropanoid char.

Increasing the heating rate displaces every stage to higher temperature and narrows the apparent reaction window on a time basis (Figure 3). The shift is the usual thermal-lag response of a finite particle: the center of the 0.306 mm grain lags the programmed temperature more severely as β rises, so the recorded $T_{peak}$ increases. The same lag is visible on the conversion curves, which move to the right from 20 to 80 K min^−1^ without a change in the number of steps. A three-stage oxidative sequence of this type (drying, holocellulose oxidation, and char oxidation) is that reported for pine wood [31,32], eucalyptus residues [33] and olive-tree fractions [34,35]. LTB does not show the extra low-temperature pectin peak of lemon peel [16].

Ignition and burnout temperatures and the combustibility index are listed with the stage data in Table 3 and plotted in Figure 4. $T_{i}$ increases from 484 K at 20 K min^−1^ to 522 K at 80 K min^−1^. $T_{b}$ increases from 730 K to 773 K. The index

$$S=\frac{{\left(d\alpha /dt\right)}_{max}\;{\left(d\alpha /dt\right)}_{mean}}{T_{i}^{2}\;T_{b}}$$

rises from $8.1\times 10^{-11}$ to $1.06\times 10^{-9}$ K^−3^ min^−2^ over the same interval. The increase in S is driven by the higher peak and mean rates at 60 and 80 K min^−1^, which outweigh the modest rise in $T_{i}$ and $T_{b}$. LTB therefore becomes more reactive, on an S basis, as the programmed rate rises from 20 to 80 K min^−1^, even though ignition is delayed by thermal lag. Stage 1 is moisture. Stage 2 is the holocellulose-dominated dry peak. Stage 3 is the lignin/char tail. The TGA stages therefore map onto Table 2 without residue. Moisture accounts for the first peak, holocellulose and extractives for the principal DTG maximum, and lignin plus fixed carbon for the char tail. Section 3.4 treats the conversion dependence of the apparent activation energy across those two reactive windows.

### 3.4. Kinetic Analysis

#### 3.4.1. Isoconversional Activation Energy

Apparent activation energies were calculated at α=0.1–0.9 by the Friedman (FR), Flynn–Wall–Ozawa (FWO), Kissinger–Akahira–Sunose (KAS), Starink (STK), and Vyazovkin (VY) methods. Kissinger (K) values were obtained from the two reactive DTG maxima (holocellulose and char oxidation), and are not isoconversional. The dehydration peak was not used. Linear regressions for FR, FWO, KAS, STK and K are shown in Figure 5. $E_{a}\left(\alpha \right)$ is shown in Figure 6 and listed in Table 4.

Kissinger analysis of the two reactive peaks gives $E_{a}=101.7\;k$J mol^−1^ ($R^{2}=0.9977$) for the holocellulose peaks and 272.3 kJ mol^−1^ ($R^{2}=0.866$) for char oxidation. FWO, KAS and STK agree to within 5 kJ mol^−1^ at every conversion up to α=0.7, with $R^{2}\geq 0.995$. VY lies a few kilojoules below that integral cluster. FR tracks the same rise but is systematically higher, as expected for a differential method on noisy DTG data. Mean $E_{a}$ over α=0.1–0.7 is 98–115 kJ mol^−1^, depending on the method (cross-method mean 106.4 kJ mol^−1^). That window is the holocellulose stage in Figure 3 and Table 3 (α≈0.05–0.70; mass loss 65–68 wt%; 67.53 wt% cellulose + hemicellulose). The slow rise from ~91 kJ mol^−1^ at α=0.1 to ~130 kJ mol^−1^ at α=0.7 is the shift from hemicellulose and extractives to crystalline cellulose, consistent with CrI = 52.6% and the 1737/1047 cm^−1^ FTIR bands.

At α=0.8, every integral method jumps to ~212 kJ mol^−1^ ($R^{2}\approx 0.995$) and FR to 238.7 kJ mol^−1^. That conversion sits on the char-oxidation DTG peak (Table 3; lignin 17.11 wt%; fixed carbon 19.90 wt%). The increase is therefore a change of process, not scatter. FR at α=0.8 and α=0.9 is unreliable ($R^{2}=0.75$ and 0.72). The integral methods at α=0.9 remain usable ($R^{2}\approx 0.95$), and give 141–145 kJ mol^−1^. Means used for interpretation are therefore restricted to α=0.1–0.7. The Kissinger value for the main DTG peak (101.7 kJ mol^−1^) matches the isoconversional cluster at α=0.3–0.5. The Kissinger char value (272 kJ mol^−1^) is higher than the isoconversional α=0.8 cluster, and rests on a weak three-point fit ($R^{2}=0.866$); it is reported, but is not used as the design $E_{a}$ for that stage.

LTB therefore has a low-barrier holocellulose stage (~100–115 kJ mol^−1^) and a high-barrier char stage (~210 kJ mol^−1^). That split is the kinetic counterpart of the two reactive DTG peaks and of the fiber composition in Table 2. After drying, the same two reactive windows (holocellulose, then char) are reported for pine and eucalyptus wood under oxidative TGA [31,32,33]. Lemon peel under the same heating rates reaches a higher peak $E_{a}$ (~304 kJ mol^−1^) because the late stage is a different tissue [16].

#### 3.4.2. Reaction Mechanism: Criado Master Plots and Šesták–Berggren Fit

Normalized Criado $z\left(\alpha \right)/z\left(0.5\right)$ curves at 20, 40, 60 and 80 K min^−1^ are compared with the theoretical F, D, A and R models in Figure 7. A single-event Šesták–Berggren function (Equation (9)) was fitted to the experimental master curve (Figure 8). The fitted exponents are m=0.174 and n=2.3, with $R^{2}=0.890$.

From α=0.1 to α=0.5, the four experimental traces overlie one another and follow the second- and third-order (F2, F3) and three-dimensional diffusion (D3) families. That interval is the stage 2 peak in Figure 3 (α≈0.05–0.70; Table 3) and the isoconversional window of 98–115 kJ mol^−1^ in Table 4. A reaction order near 2, with a weak nucleation term (m=0.174), is consistent with overlapping depolymerization of the holocellulose fraction (hemicellulose 15.42 wt% and cellulose 52.11 wt%) in a moderately crystalline wall (CrI = 52.6%). The SEM images (Figure 2) show milled fibres, rather than a dense compact, so a mixed F/D description is physically reasonable. These assignments are compositional correspondences, and stage 2 is not resolved into named polymer sub-peaks. Beyond α=0.5, the experimental $z\left(\alpha \right)$ falls below every ideal model, reaching ~0.26 at α=0.7, then scatters at α=0.8–0.9 where the four heating rates no longer coincide. That break is the start of char oxidation (Table 3; lignin 17.11 wt%; fixed carbon 19.90 wt%; $E_{a}\approx 212\;k$J mol^−1^ at α=0.8). A single theoretical $f\left(\alpha \right)$ cannot describe both windows. The single-event SB function reproduces the master curve through the stage 2 ($R^{2}=0.890$) and under-predicts the late-stage decay; the residuals at α=0.10 and α=0.60–0.70 are the price of forcing one $\left(m,n\right)$ pair across a two-process DTG profile. No two-event SB model is reported. The $\left(m,n\right)$ pair is therefore an empirical descriptor of Stage 2 only. It is not used as a reactor-scale rate law, and is not applied to Stage 3. The working description of Stage 2 is F2, with D3 as the diffusion alternative indicated by Figure 7. Coats–Redfern screening of the same window is given in the next subsection.

#### 3.4.3. Coats–Redfern Screening

Sixteen solid-state models were fitted by the Coats–Redfern method to stage 2 (oxidative devolatilization) and to stage 3 (char oxidation) at each heating rate. Full $E_{a}$, $\ln A_{0}$ and $R^{2}$ values are given in Table S3. The models with $R^{2}\geq 0.995$ on stage 2 are summarized in Table 5.

F2 gives the highest $R^{2}$ at every heating rate ($R^{2}\geq 0.9992$) and a mean $E_{a}$ of 109.0 kJ mol^−1^. That value sits inside the isoconversional cluster for α=0.1–0.7 (Table 4: FWO 109.2, KAS 104.7, STK 105.1, FR 115.3 kJ mol^−1^) and matches the Kissinger stage 2 peak (101.7 kJ mol^−1^). F3 returns a similar $R^{2}$, but a negative $\ln A_{0}$ and an $E_{a}$ (~146 kJ mol^−1^) above the isoconversional mean; it is rejected. D3 remains a plausible second choice ($R^{2}\geq 0.9966$, mean $E_{a}$ 148 kJ mol^−1^), and is consistent with the Criado overlay on F2/F3/D3 up to α=0.5 (Figure 8). The working model for stage 2 is therefore F2, with D3 as the diffusion alternative indicated by the master plot. Coats–Redfern on the char-oxidation window (stage 3) is not stable. Apparent $E_{a}$ for a given model falls as the heating rate rises (F2: 243 → 65 kJ mol^−1^ from 20 to 80 K min^−1^; D3: 133 → 49 kJ mol^−1^). Several nucleation and power-law fits produce negative $E_{a}$. High $R^{2}$ at a single heating rate does not identify a mechanism under those conditions. The char step is therefore described by the isoconversional jump at α=0.8 (~212 kJ mol^−1^, Table 4) and by the break in the Criado plot after α=0.5, not by a CR triplet.

#### 3.4.4. Kinetic Compensation Effect

The kinetic compensation effect was tested on the Friedman pair $\left(E_{a},\ln A_{0}\right)$ at α=0.1–0.5, the conversion range in which Criado and the isoconversional methods describe a single holocellulose process. The plot is linear (Figure 9): $\ln A_{0}=0.1563\;E_{a}+3.4784\;\left(R^{2}=0.9954\right)$, with $E_{a}$ in kJ mol^−1^ and $A_{0}$ in min^−1^. The isokinetic temperature is $T_{iso}=\frac{1}{bR}=770\;K.$ The five points used are α=0.1–0.5 ($E_{a}=96.14$–116.21 kJ mol^−1^; $\ln A_{0}=18.42$–21.58). Linearity in that window indicates that the rise in $E_{a}$ through the holocellulose peak is a compensated change in $A_{0}$, not a numerical artefact. $T_{iso}=770$ K lies above the holocellulose DTG maxima (600–640 K, Table 3) and inside the char-oxidation window, which is consistent with a change of rate-limiting step after the main peak. The compensation relation is not extended to α=0.8–0.9, where $E_{a}$ and the master plot both change process.

### 3.5. Thermodynamic Parameters

Enthalpy (ΔH), Gibbs free energy (ΔG) and entropy (ΔS) of activation were calculated from the isoconversional $E_{a}$ and $A_{0}$ at the mean DTG peak temperature of the relevant stage: $T_{p}=620.3$ K for α=0.1–0.7 and $T_{p}=712.0$ K for α=0.8–0.9. Values from the integral methods (FWO, KAS, and STK) and from Friedman are given in Table 6.

$\Delta H=E_{a}-RT_{p}$ lies 5–6 kJ mol^−1^ below $E_{a}$ at every conversion. The gap is the work of expansion to the activated state, and is the magnitude expected at 620–712 K. Through the holocellulose window, ΔH rises from ~86–91 kJ mol^−1^ at α=0.1 to ~126–143 kJ mol^−1^ at α=0.7, in step with Table 4. At α=0.8, ΔH jumps to ~206 kJ mol^−1^ (integral methods), the thermodynamic counterpart of the char-oxidation barrier. ΔG is positive at every conversion (169–214 kJ mol^−1^). The activated complex is therefore not in spontaneous equilibrium with the reactant at $T_{p}$; an external heat flux is required, as in any combustion TGA experiment. Integral-method ΔG is lower for FWO (~169–184 kJ mol^−1^) than for KAS/STK/FR (~189–214 kJ mol^−1^) because FWO returns a larger $A_{0}$. The ranking does not change the sign or the conclusion. ΔS is negative through α=0.1–0.7 (−0.07 to −0.18 kJ mol^−1^ K^−1^). The activated complex is more ordered than the reactant, which is consistent with a bimolecular or diffusion-assisted step on a fiber surface (F2/D3, Figure 7). At α=0.8, ΔS approaches zero or becomes slightly positive, in line with a different, higher-energy char step. Friedman values at α=0.9 are omitted; $E_{a}=51.3\;k$J mol^−1^ and $R^{2}=0.72$ are not a physical tail.

The thermodynamic set, therefore, restates the kinetic split already fixed by Table 4, Figure 3 and Figure 7: a feasible, entropy-decreasing holocellulose stage at ΔH≈90–130 kJ mol^−1^, followed by a higher-ΔH char stage near 206 kJ mol^−1^. Combined with HHV = 18.50 MJ kg^−1^ and ash = 2.30 wt% (Table 2), the numbers describe a woody pruning fuel that ignites on the holocellulose peak and finishes on a slower char tail.

### 3.6. Integrated Analysis and Implications for Biofuel Applications

This study shows that lemon-tree branch is a viable woody solid fuel whose composition, combustion profile and kinetic parameters are those of lignified pruning wood, rather than of fruit-processing residues. The higher heating value is 18.50 ± 0.25 MJ kg^−1^, which lies next to lemon-orchard pellets (18.60 MJ kg^−1^; [9]) and above Spanish lemon-tree branches (17.56 MJ kg^−1^; [10]) and the mean calorific value of orange and mandarin wood (17.4 MJ kg^−1^; [5]). Combined with a volatile-matter content of 77.80 ± 0.35 wt% and a fixed-carbon content of 19.90 ± 0.25 wt%, this energy density is sufficient for pelletizing or co-firing after drying below 10 wt% moisture.

Ash is 2.30 ± 0.10 wt%, comparable to citrus pruning (1.80 wt%; [11]) and well below lemon-orchard pellets (5.20 wt%; [9]) and lemon peel [16]. EDX on the washed powder detects calcium as the only quantified inorganic cation, and does not detect potassium. Because the wood was rinsed, this K-poor surface is reported for the washed fuel and is not claimed for unwashed orchard residues. Nitrogen (0.55 ± 0.04 wt%) and Sulphur (0.18 ± 0.02 wt%) imply a limited fuel-NO_x_ and SO_2_ inventory, consistent with the low SO_2_ reported for orange-pruning combustion in a fluidized bed [3].

Oxidative TGA at 20–80 K min^−1^ resolves three stages: dehydration, holocellulose devolatilization (65–68 wt% loss) and char oxidation (25 wt% loss). Mean isoconversional activation energies on α=0.1–0.7 are 98–115 kJ mol^−1^. The barrier rises to approximately 212 kJ mol^−1^ at α=0.8, coinciding with the char peak. Criado master plots follow F2, F3 and D3 up to α=0.5. A single-event Šesták–Berggren function with m=0.174 and n=2.3 is an empirical fit for Stage 2 only ($R^{2}=0.890$). Coats–Redfern selects the second-order model F2 for the holocellulose stage (mean $E_{a}=109.0\;k$J mol^−1^). The char-stage Coats–Redfern triplet is not stable with heating rate, and is not used for design. The combustibility index increases from $8.14\times 10^{-11}$ to $1.06\times 10^{-9}$K^−3^ min^−2^ between 20 and 80 K min^−1^, indicating higher apparent reactivity as the programmed rate rises from 20 to 80 K min^−1^, while ignition is delayed from 484 K to 522 K by thermal lag.

For boiler or pellet use, three practical constraints follow. First, the low ash and Ca-dominated inorganic fraction reduce the need for aggressive soot-blowing relative to herbaceous or peel fuels, but grit handling is still required. Second, the particle size used here (0.306 mm) is already in the range of pulverized biomass; further milling is justified only if a diffusion contribution becomes limiting in a thick char layer. Third, the combustor must retain the charge above approximately 750 K long enough to finish the lignin/char tail. On that basis, LTB is recommended as a dried pruning fuel for pellets or co-firing, not as a high-HHV substitute for citrus peel.

## 4. Conclusions

Lemon-tree pruning wood was characterized and combusted at 20–80 K min^−1^. The solid is a moderately crystalline xylem (cellulose 52.11 wt%, CrI 52.6%, lignin 17.11 wt%) with moisture 7.10 wt%, volatile matter 77.80 wt%, fixed carbon 19.90 wt%, ash 2.30 wt% and HHV 18.50 MJ kg^−1^. Nitrogen and Sulphur are 0.55 and 0.18 wt%. FTIR, XRD and SEM–EDX agree with that assignment, and show a Ca-bearing surface on the washed fuel. Combustion proceeds in three stages: dehydration, holocellulose devolatilization (65–68 wt% loss, 600–640 K) and char oxidation (25 wt% loss, 710–730 K). Mean isoconversional $E_{a}$ on α=0.1–0.7 is 98–115 kJ mol^−1^ (FWO 109.2, KAS 104.7, STK 105.1, FR 115.3 kJ mol^−1^). At α=0.8, the integral methods rise to ~212 kJ mol^−1^. Kissinger values are 101.7 and 272.3 kJ mol^−1^ for the two DTG peaks. Criado master plots follow F2/F3/D3 up to α=0.5. A single-event Šesták–Berggren function with m=0.174 and n=2.3 is an empirical fit for Stage 2 only ($R^{2}=0.890$). Coats–Redfern selects F2 on the holocellulose stage (mean $E_{a}=109.0\;k$J mol^−1^), and does not yield a stable char triplet. The Friedman compensation relation on α=0.1–0.5 is $\ln A_{0}=0.1563E_{a}+3.4784$ ($R^{2}=0.9954$), with $T_{iso}=770\;$K. ΔH tracks $E_{a}$ by ~5 kJ mol^−1^, ΔG remains positive (169–214 kJ mol^−1^), and ΔS is negative on the holocellulose stage. $T_{i}$ increases from 484 K to 522 K and S from $8.14\times 10^{-11}$ to $1.06\times 10^{-9}$ K^−3^ min^−2^ as the heating rate rises from 20 to 80 K min^−1^. LTB is a low-ash, low-S woody biofuel with an established citrus-pruning heating value and a two-barrier kinetic scheme. It is suitable for pelletizing, or for co-firing provided the combustor finishes the char above ~750 K. The kinetic and thermodynamic parameters reported here apply to LTB combustion at 20–80 K min^−1^, and could be used in the design of pellet and co-firing systems.

## Figures and Tables

**Figure 1:**
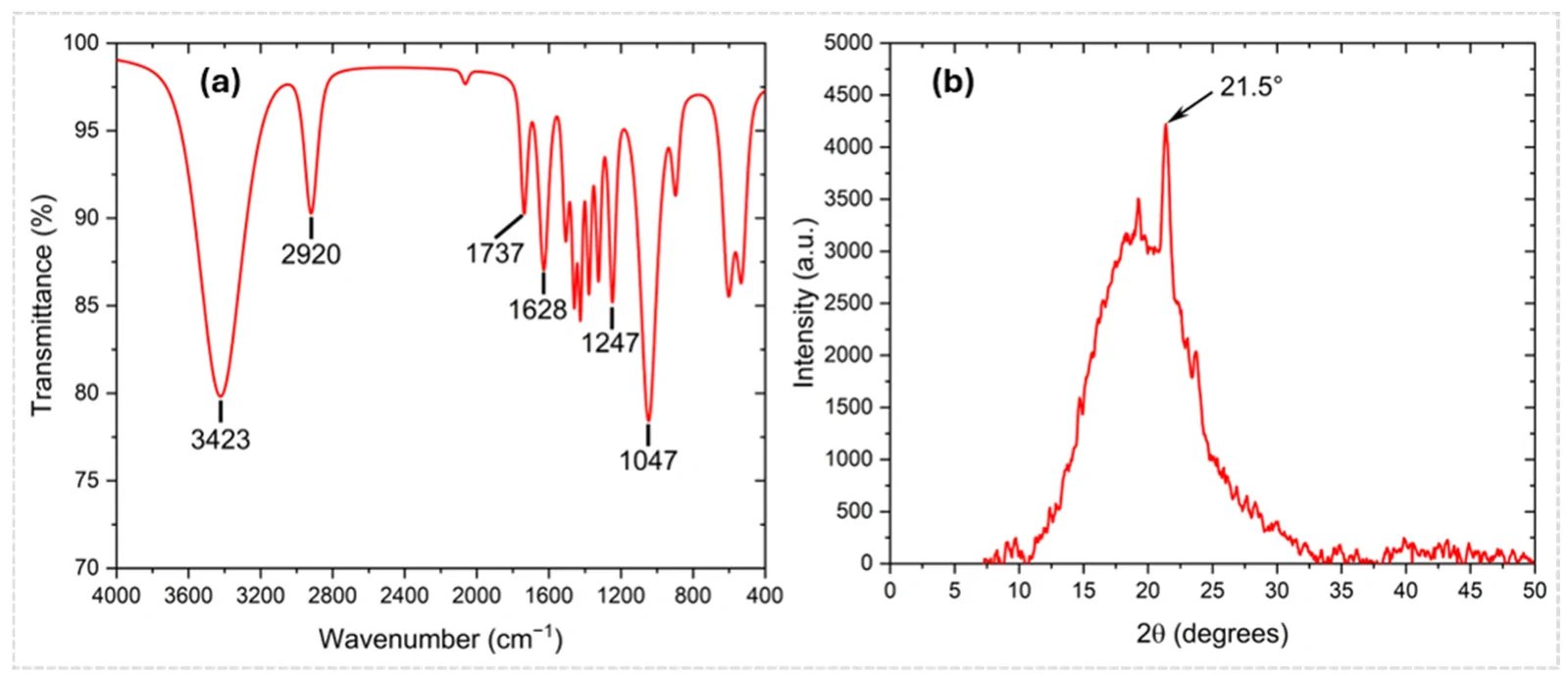
(**a**) FTIR transmission spectrum of LTB (KBr). (**b**) Powder XRD pattern; the cellulose I (002) maximum is marked at 2θ=21.5°.

**Figure 2:**
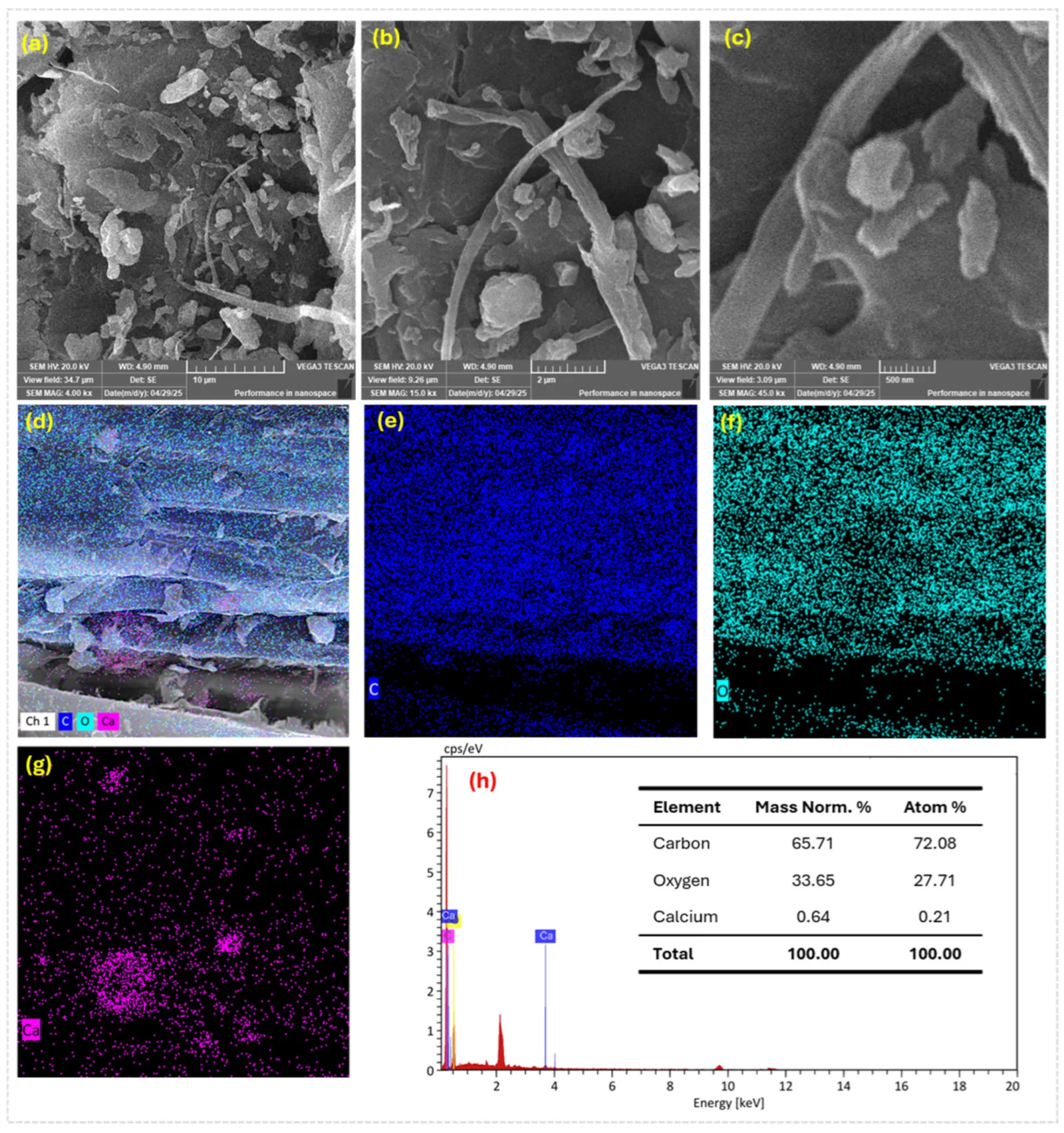
SEM–EDX of LTB: (**a**–**c**) secondary-electron images at increasing magnification (scale bars: 10 µm, 2 µm, and 500 nm); (**d**) combined elemental map (carbon in blue, oxygen in cyan, and calcium in magenta); (**e**) carbon map (blue); (**f**) oxygen map (cyan); (**g**) calcium map (magenta); (**h**) EDX spectrum and quantification table.

**Figure 3:**
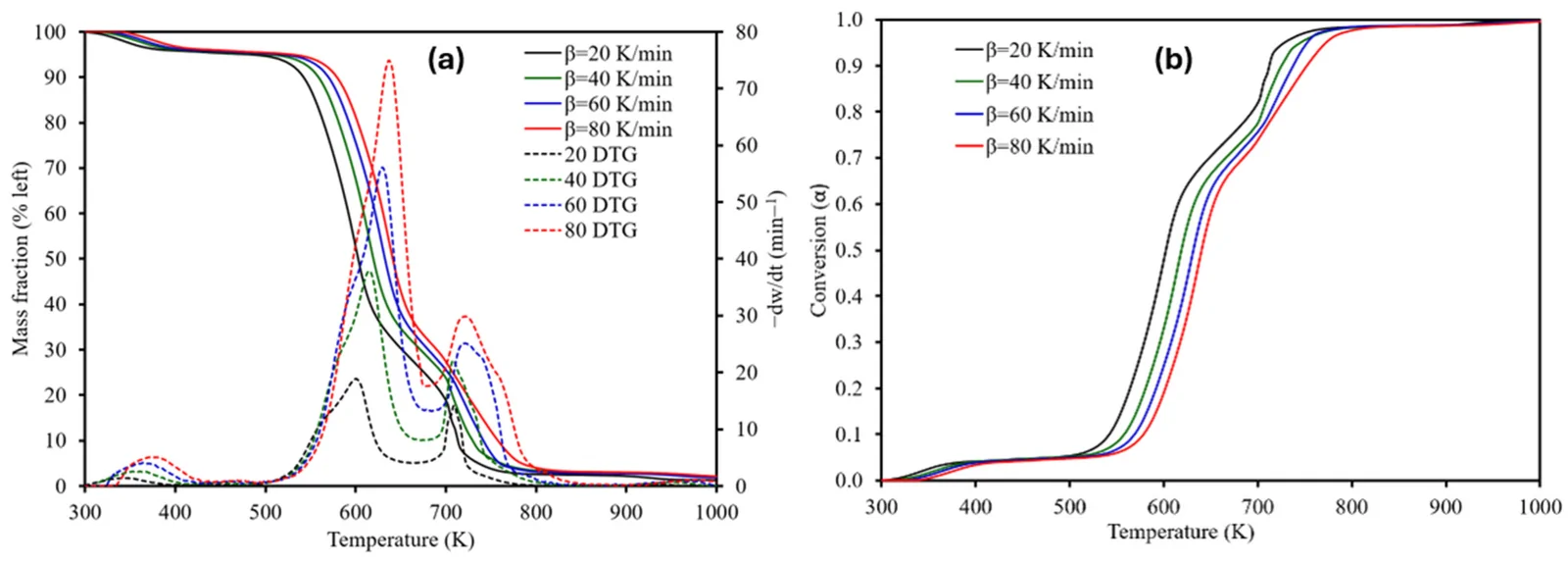
Combustion of LTB at 20, 40, 60 and 80 K min^−1^: (**a**) TG and DTG curves; (**b**) conversion α versus temperature.

**Figure 4:**
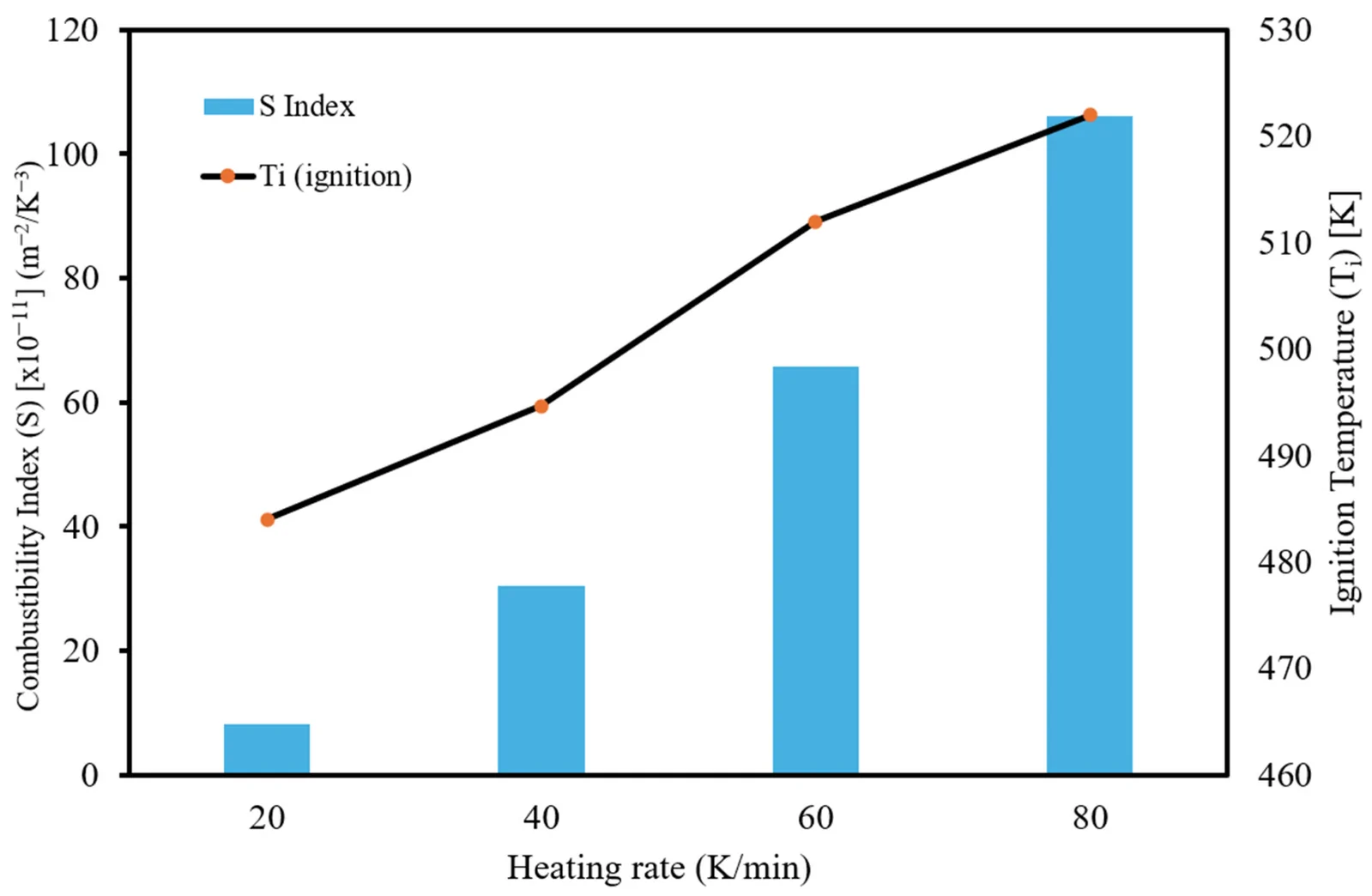
Combustibility index S and ignition temperature $T_{i}$ of LTB as a function of heating rate.

**Figure 5:**
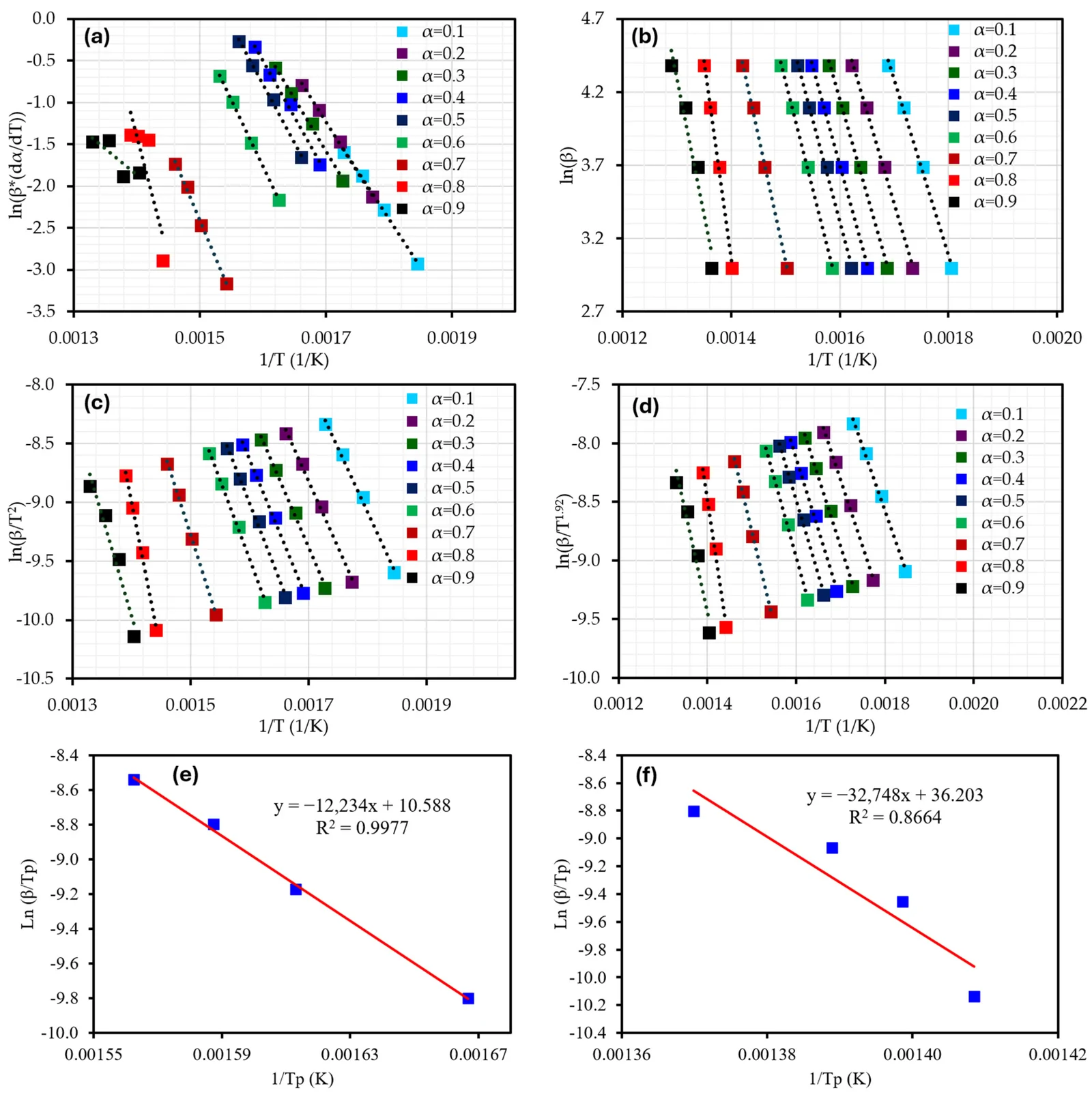
Isoconversional regressions for LTB combustion: (**a**) Friedman; (**b**) FWO; (**c**) KAS; (**d**) Starink; (**e**,**f**) Kissinger plots of the holocellulose and char-oxidation DTG peaks. (Black dotted lines indicate linear regression fits across heating rates at fixed conversion values; blue square markers in (**e**) and (**f**) indicate experimental peak points, and red solid lines indicate the corresponding linear Kissinger fits).

**Figure 6:**
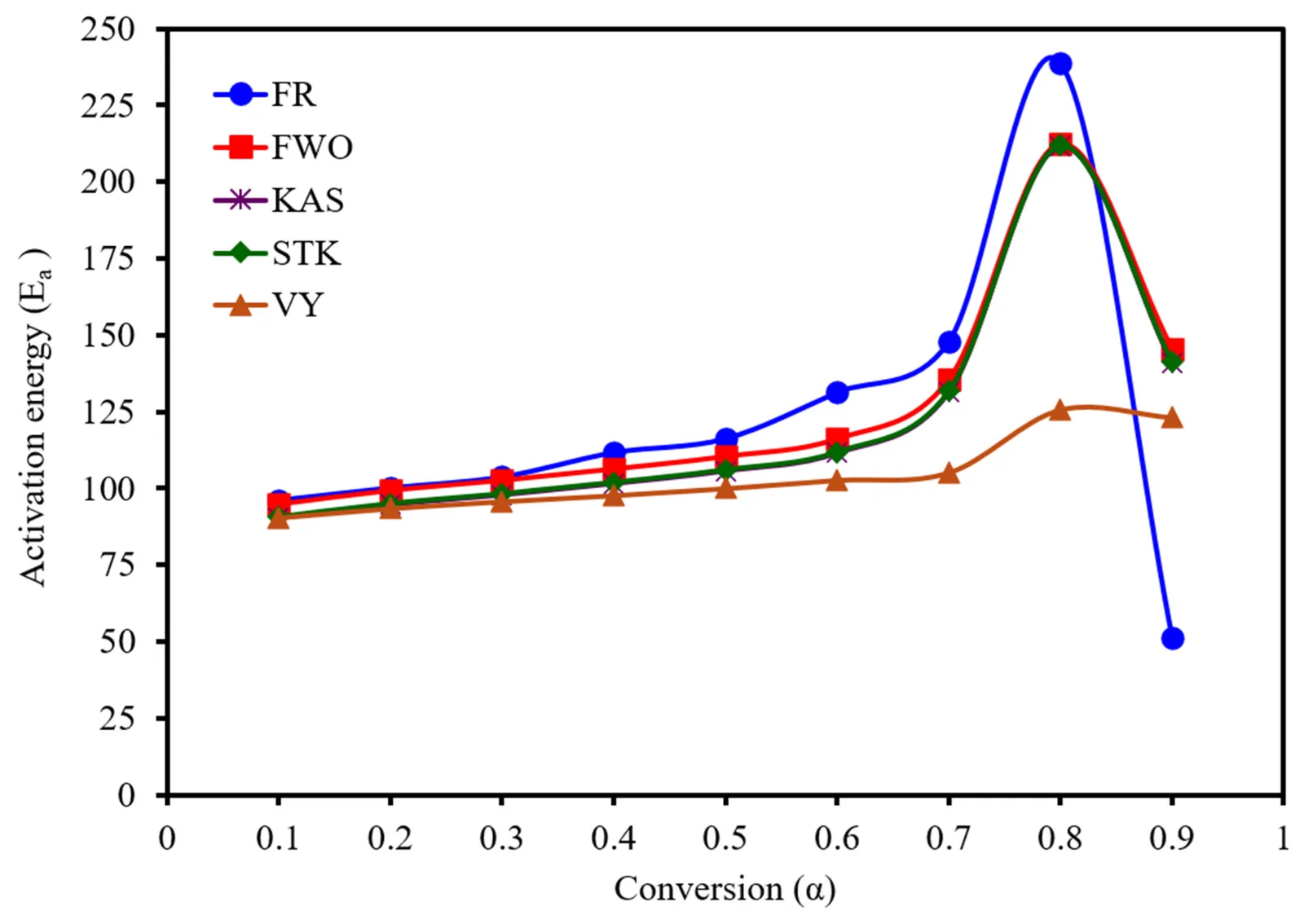
Apparent activation energy versus conversion from FR, FWO, KAS, STK and VY.

**Figure 7:**
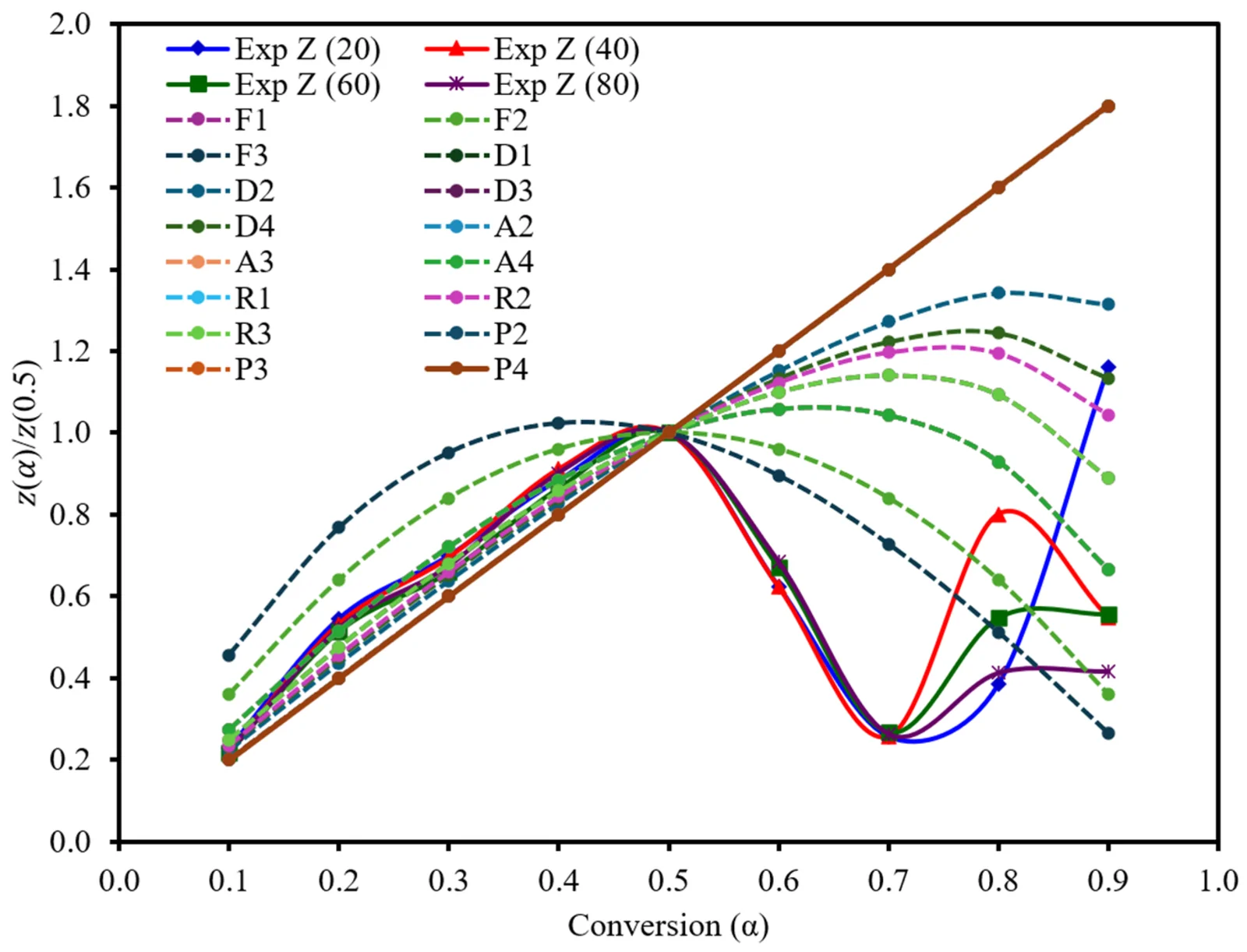
Criado master plots of LTB at 20–80 K min^−1^ and theoretical $z\left(\alpha \right)/z\left(0.5\right)$ curves.

**Figure 8:**
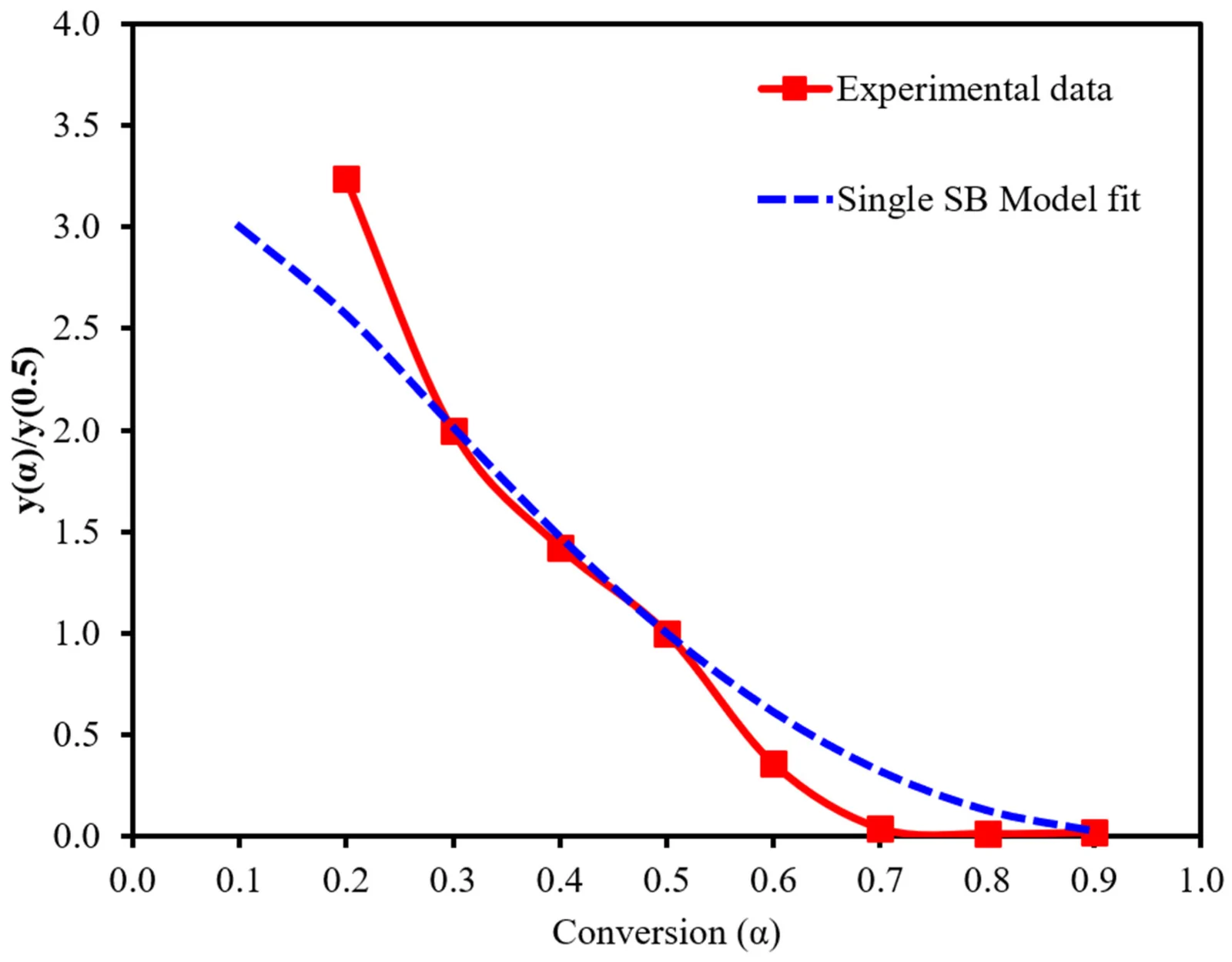
Single-event Šesták–Berggren fit to the experimental master curve.

**Figure 9:**
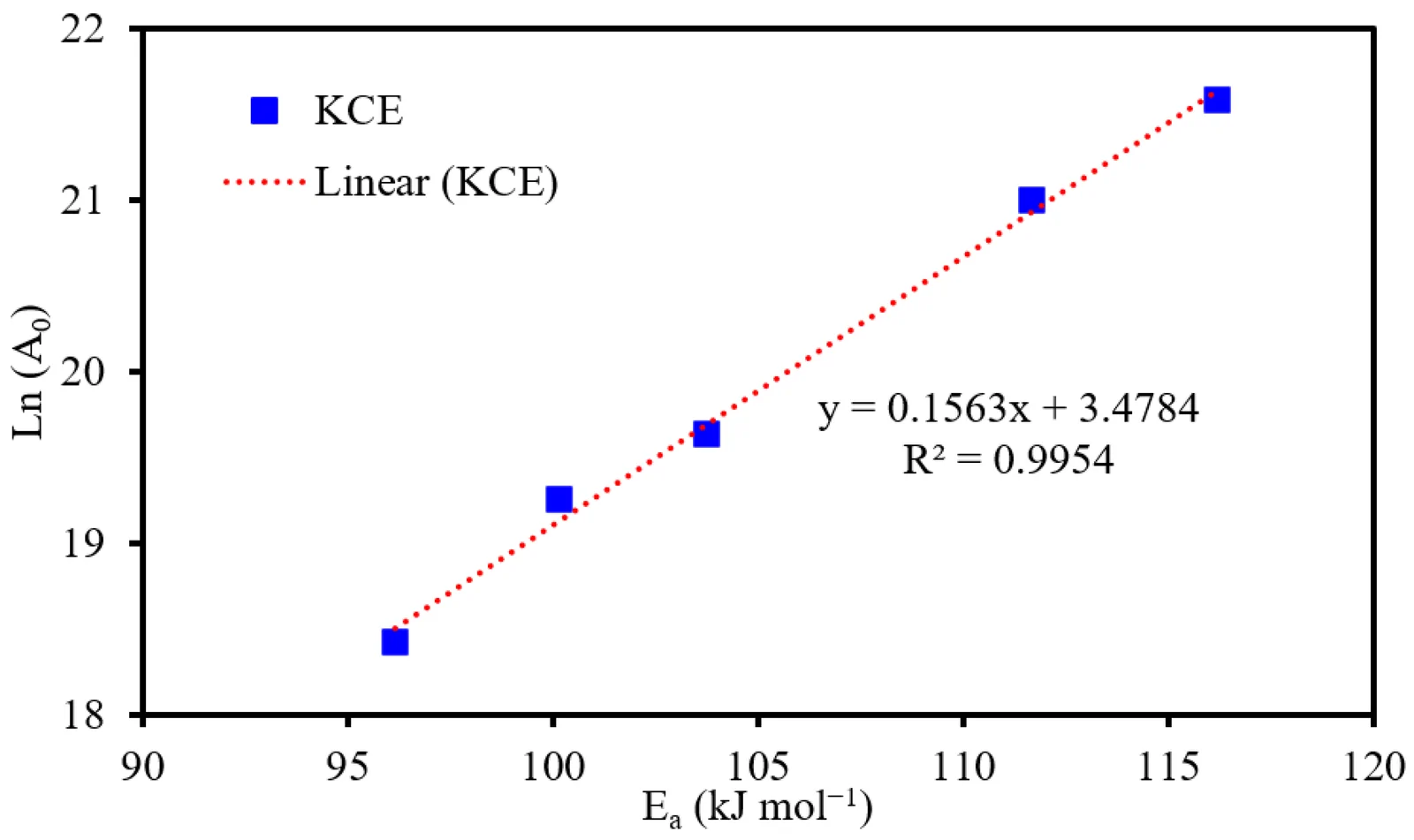
Kinetic compensation plot of $\ln A_{0}$ versus $E_{a}$ from the Friedman method at α=0.1–0.5.

**Table 1:** Analytical framework for the physicochemical characterization of lemon-tree branch (LTB).

Property	Method/Principle	Instrument	Operating Conditions
Proximate analysis (moisture, volatile matter, ash, fixed carbon)	High-resolution TGA aligned with ASTM E1131 [24]	TGA-2 Star System (Mettler Toledo, Greifensee, Switzerland)	383 K, 30 min, $N_{2}$; 1173 K, 7 min, $N_{2}$; 823 K, 15 min, $O_{2}$
Ultimate analysis (C, H, N, S; O by difference)	Flash combustion, ASTM D5373 [25]	Vario EL III (Elementar Analysensysteme GmbH, Langenselbold, Germany)	$O_{2}$, 1273 K
Higher heating value	Isoperibol bomb calorimetry, ASTM D5865 [26]	Parr 1341EE (Parr Instrument Company, Moline, IL, USA)	3.0 MPa $O_{2}$; benzoic acid calibration
Fiber fractions (NDF, ADF, ADL)	Van Soest sequential digestion	ANKOM 2000 (ANKOM Technology, Macedon, NY, USA)	Neutral detergent; acid detergent; $72\%{\;H}_{2}{\mathrm{SO}}_{4}$

**Table 2:** Proximate, ultimate, fiber and calorific composition of lemon-tree branch (LTB). Moisture is as received; remaining values are dry basis. Mean ± SD, *n* = 3.

Analysis	Parameter	LTB
Proximate analysis	Moisture (as received, wt%)	7.10 ± 0.15
	Volatile matter (wt%)	77.80 ± 0.35
	Fixed carbon (wt%)	19.90 ± 0.25
	Ash (wt%)	2.30 ± 0.10
Ultimate analysis	C (wt%)	48.30 ± 0.20
	H (wt%)	5.90 ± 0.12
	N (wt%)	0.55 ± 0.04
	S (wt%)	0.18 ± 0.02
	O (wt%)	42.77 ± 0.22
Fiber fractions	Cellulose (wt%)	52.11 ± 0.35
	Hemicellulose (wt%)	15.42 ± 0.50
	Lignin (wt%)	17.11 ± 0.11
	Extractives (wt%)	15.36 ± 0.28
Calorific value	HHV (MJ kg^−1^)	18.50 ± 0.25
	HHV (Cal g^−1^)	4422 ± 60

**Table 3:** Combustion stages, ignition and burnout temperatures, and combustibility index of LTB.

β (K min^−1^)	Stage	$T_{range}$ (K)	$T_{peak}$ (K)	Mass Loss (wt%)	Peak Rate (wt% min^−1^)	$T_{i}$ (K)	$T_{b}$ (K)	S (K^−3^ min^−2^)
20	Stage 1—Dehydration	300–400	350	5	2	484	730	$8.14\times 10^{-11}$
	Stage 2—Devolatilization	500–650	600	65	19			
	Stage 3—Char oxidation	680–750	710	25	13			
40	Stage 1—Dehydration	320–400	355	5	3	495	747	$3.04\times 10^{-10}$
	Stage 2—Devolatilization	500–670	620	65	38			
	Stage 3—Char oxidation	680–770	715	25	23			
60	Stage 1—Dehydration	330–410	370	5	3	512	754	$6.58\times 10^{-10}$
	Stage 2—Devolatilization	520–680	630	67	58			
	Stage 3—Char oxidation	690–780	720	25	25			
80	Stage 1—Dehydration	340–420	375	5	4	522	773	$1.06\times 10^{-9}$
	Stage 2—Devolatilization	550–690	640	68	77			
	Stage 3—Char oxidation	690–800	730	25	16			

**Table 4:** Apparent activation energy ($E_{a}$, kJ mol^−1^) and coefficient of determination ($R^{2}$) for LTB at α=0.1–0.9.

	FR		FWO		KAS		STK		VY		Mean	
α	E_a_	R^2^	E_a_	R^2^	E_a_	R^2^	E_a_	R^2^	E_a_	R^2^	E_a_	R^2^
0.1	96.1	0.9982	94.7	0.9966	90.4	0.9958	90.7	0.9958	90.5	NA	92.5	0.9966
0.2	100.2	0.998	99.2	0.9970	94.7	0.9962	95.0	0.9962	93.5	NA	96.5	0.9969
0.3	103.7	0.9969	102.5	0.9974	97.9	0.9968	98.2	0.9968	95.8	NA	99.6	0.9970
0.4	111.7	0.9948	106.2	0.9974	101.6	0.9967	101.9	0.9968	97.8	NA	103.8	0.9964
0.5	116.2	0.9971	110.3	0.9968	105.7	0.9961	106.0	0.9961	100.1	NA	107.7	0.9965
0.6	131.3	0.9997	116.0	0.9984	111.5	0.998	111.9	0.9980	102.8	NA	114.7	0.9985
0.7	147.9	0.9958	135.4	0.9983	131.3	0.998	131.7	0.9980	105.2	NA	130.3	0.9975
0.8	238.7	0.7476	212.3	0.9955	211.6	0.9949	211.9	0.9950	125.7	NA	200.0	0.9333
0.9	51.3	0.7244	145.4	0.9583	140.8	0.9509	141.2	0.9512	123.2	NA	120.4	0.8962
Mean (0.1–0.7)	115.3	0.9972	109.2	0.9974	104.7	0.9968	105.1	0.9968	97.9	—	106.4	0.9971

NA: $R^{2}$ is not defined for the non-linear Vyazovkin method.

**Table 5:** Coats–Redfern parameters for the holocellulose stage of LTB.

Model	20 K min^−1^ $E_{a}$/$\ln A_{0}$/$R^{2}$	40 K min^−1^ $E_{a}$/$\ln A_{0}$/$R^{2}$	60 K min^−1^ $E_{a}$/$\ln A_{0}$/$R^{2}$	80 K min^−1^ $E_{a}$/$\ln A_{0}$/$R^{2}$	Mean $E_{a}$ (kJ mol^−1^)
F1	71.9/10.9/0.9985	78.5/10.8/0.9984	78.8/11.5/0.9981	82.5/11.4/0.9974	77.9
F2	101.4/5.0/0.9996	108.6/4.9/0.9995	111.6/5.2/0.9995	114.5/5.3/0.9992	109.0
F3	136.5/−2.2/0.9996	144.2/−2.1/0.9996	150.7/−2.3/0.9997	152.3/−1.9/0.9993	145.9
D3	136.7/1.4/0.9981	149.6/0.4/0.9980	149.2/1.5/0.9976	157.1/0.8/0.9966	148.2

**Table 6:** Pre-exponential factor and thermodynamic parameters of LTB. ΔH and ΔG are in kJ mol^−1^; ΔS is in kJ mol^−1^ K^−1^, and $A_{0}$ (min^−1^).

	FR					FWO				
α	$R^{2}$	$A_{0}$	ΔH	ΔG	ΔS	$R^{2}$	$A_{0}$	ΔH	ΔG	ΔS
0.1	0.9982	$2.48\times 10^{6}$	91.0	197.0	−0.1709	0.9966	$1.07\times 10^{8}$	89.6	176.2	−0.1396
0.2	0.9980	$1.27\times 10^{7}$	95.0	192.6	−0.1573	0.9970	$5.08\times 10^{8}$	94.1	172.6	−0.1267
0.3	0.9969	$3.20\times 10^{7}$	98.6	191.4	−0.1497	0.9974	$1.38\times 10^{9}$	97.3	170.8	−0.1184
0.4	0.9948	$1.93\times 10^{8}$	106.5	190.1	−0.1347	0.9974	$3.60\times 10^{9}$	101.1	169.5	−0.1104
0.5	0.9971	$5.16\times 10^{8}$	111.0	189.6	−0.1266	0.9968	$9.49\times 10^{9}$	105.1	168.6	−0.1023
0.6	0.9997	$5.23\times 10^{9}$	126.2	192.7	−0.1073	0.9984	$2.91\times 10^{10}$	110.9	168.6	−0.0930
0.7	0.9958	$1.70\times 10^{10}$	142.7	203.2	−0.0975	0.9983	$4.98\times 10^{11}$	130.2	173.3	−0.0694
0.8	0.7476	$5.68\times 10^{16}$	232.8	214.1	0.0262	0.9955	$1.13\times 10^{17}$	206.4	183.6	0.0320
0.9	0.7244	$1.45\times 10^{3}$	45.4	212.0	−0.2340	0.9583	$7.58\times 10^{11}$	139.5	187.2	−0.0671
	KAS					STK				
α	$R^{2}$	$A_{0}$	ΔH	ΔG	ΔS	$R^{2}$	$A_{0}$	ΔH	ΔG	ΔS
0.1	0.9958	$4.61\times 10^{5}$	85.2	199.9	−0.1849	0.9958	$8.32\times 10^{5}$	85.5	197.2	−0.1800
0.2	0.9962	$2.23\times 10^{6}$	89.5	196.1	−0.1718	0.9962	$4.03\times 10^{6}$	89.9	193.4	−0.1669
0.3	0.9968	$6.12\times 10^{6}$	92.7	194.1	−0.1634	0.9968	$1.11\times 10^{7}$	93.1	191.4	−0.1585
0.4	0.9967	$1.65\times 10^{7}$	96.4	192.7	−0.1552	0.9968	$2.99\times 10^{7}$	96.8	190.0	−0.1502
0.5	0.9961	$4.54\times 10^{7}$	100.6	191.6	−0.1468	0.9961	$8.24\times 10^{7}$	100.9	188.8	−0.1418
0.6	0.9980	$1.48\times 10^{8}$	106.4	191.3	−0.1369	0.9980	$2.70\times 10^{8}$	106.7	188.6	−0.1320
0.7	0.9980	$3.16\times 10^{9}$	126.2	195.3	−0.1115	0.9980	$5.77\times 10^{9}$	126.5	192.6	−0.1065
0.8	0.9949	$1.60\times 10^{15}$	205.7	208.1	−0.0034	0.9950	$2.94\times 10^{15}$	206.0	204.8	0.0016
0.9	0.9509	$4.58\times 10^{9}$	134.9	212.9	−0.1096	0.9512	$8.43\times 10^{9}$	135.3	209.7	−0.1045

## Data Availability

The original contributions presented in this study are included in the Article/Supplementary Material. Further inquiries can be directed to the corresponding author.

## Supplementary Materials

The following supporting information can be downloaded at: https://www.mdpi.com/article/10.3390/polym18192344/s1. Table S1: Working equations and linear regression variables of the five isoconversional kinetic methods (Friedman, Flynn–Wall–Ozawa, Kissinger–Akahira–Sunose, Starink, and Vyazovkin) [18,19]; Table S2: Differential $f\left(\alpha \right)$ and integral $g\left(\alpha \right)$ reaction functions of the sixteen solid-state mechanistic models [18,21]; Table S3: Apparent activation energies, pre-exponential factors, and coefficients of determination from Coats–Redfern screening of Stage 2 and Stage 3 across heating rates (20, 40, 60, and 80 K min^−1^) [29].
