# Supplementary material for: Combustion Kinetics of Natural Polymers in Lemon-Tree Pruning Wood at High Heating Rates: Criado Master Plots and Šesták–Berggren Modelling
Source: Polymers (Basel). 2026 Sep 25;18(19):2344. doi: 10.3390/polym18192344 (PMC13666915; doi:10.3390/polym18192344)
Supplement: Supplementary file 1 [file polymers-18-02344-s001.zip › polymers-4590797-supplementary.pdf]

polymers

MDPI

Supplementary Materials for

Combustion Kinetics of Natural Polymers in Lemon-Tree Pruning Wood at High Heating Rates: Criado Master Plots and Šesták–Berggren Modelling

Mohamed Anwar Ismail [1], Ibrahim Dubdub [2,*], Suleiman Mousa [2] and Mohammed Al-Yaari [2]

[1] Mechanical Engineering Department, College of Engineering, King Faisal University, P.O. Box 380, Al-Ahsa 31982, Saudi Arabia; maismail@kfu.edu.sa (M.A.I.)
[2] Chemical Engineering Department, College of Engineering, King Faisal University, P.O. Box 380, Al-Ahsa 31982, Saudi Arabia; saamousa@kfu.edu.sa (S.M.); malyaari@kfu.edu.sa (M.A.-Y.)
* Correspondence: idubdub@kfu.edu.sa (I.D.); Tel.: +966-13-5896989

Table S1. Working equations and linear regression variables of the five isoconversional kinetic methods and the Coats–Redfern model-fitting method.

model-free methods		
**Method**	**Formula**	**Plot**
FR	$ln\left(\beta\frac{d\alpha}{dT}\right) = ln[A_o f(\alpha)] - \frac{E_a}{RT}$ (1)	$ln\left(\beta\frac{d\alpha}{dT}\right) vs. \frac{1}{T}$
FWO	$ln(\beta) = ln\frac{A_o E_a}{Rg(\alpha)} - 5.331 - 1.052\frac{E_a}{RT}$ (2)	$ln(\beta)\ vs.\ \frac{1}{T}$
KAS	$ln\left(\frac{\beta}{T^2}\right) = ln\frac{A_o R}{E_a\, g(\alpha)} - \frac{E_a}{RT}$ (3)	$ln\left(\frac{\beta}{T^2}\right) vs. \frac{1}{T}$
STK	$ln\frac{\beta}{T^{1.92}} = ln\left(\frac{A_0 E_a}{Rg(\alpha)}\right) - 1.0008\frac{E_a}{RT}$ (4)	$ln\frac{\beta}{T^{1.92}}\ vs.\ \frac{1}{T}$
K	$\ln(\frac{\beta}{T_m^2}) = ln\left(\frac{A_0 R}{E_a}\right) - \frac{E_a}{RT_m}$ (5)	$\ln(\frac{\beta}{T_m^2})\ vs.\ \frac{1}{T_m}$
VY	$\Phi(E_\alpha) = \sum_{i=1}^{n}\sum_{j\neq i}^{n}\frac{J[E_\alpha, T_i(t_\alpha)]}{J[E_\alpha, T_j(t_\alpha)]} = 0$ (6)	minimizing the function $\Phi(E_\alpha)$
model-fitting methods		
**Method**	**Formula**	**Plot**
CR	$ln\left[\frac{g(\alpha)}{T^2}\right] = ln\left[\frac{A_o R}{\beta E_a}\right] - \frac{E_a}{RT}$ (7)	$ln\left[\frac{g(\alpha)}{T^2}\right] vs. \frac{1}{T}$

Methods and working equations compiled from ICTAC Kinetics Committee Recommendations [18,19] and Coats and Redfern [29]. Symbols are defined in Section 2.5 of the main text.

Table S2. Differential $f(\alpha)$ and integral $g(\alpha)$ reaction functions of the sixteen solid-state mechanistic models.

**Reaction mechanism**	**Code**	$\boldsymbol{f(\alpha)}$	$\boldsymbol{g(\alpha)}$
Reaction order models-1st order	F1	1-α	$-\ln(1-\alpha)$
Reaction order models-2ndorder	F2	$(1-\alpha)^2$	$(1-\alpha)^{-1}-1$
Reaction order models-3rd order	F3	$(1-\alpha)^3$	$[(1-\alpha)^{-2}-1]/2$
Diffusion model-1 dimension	D1	$1/2\alpha^{-1}$	α^2
Diffusion model-2-dimension	D2	$[-ln(1-\alpha)]^{-1}$	$(1-\alpha)\ln(1-\alpha)+\alpha$
Diffusion model-3-dimension	D3	$3/2[1-(1-\alpha)^{1/3}]^{-1}$	$[1-(1-\alpha)^{1/3}]^2$
Nucleation models-2 dimension	A2	$2(1-\alpha)[-\ln(1-\alpha)]^{1/2}$	$[-\ln(1-\alpha)]^{1/2}$
Nucleation models-3-dimension	A3	$3(1-\alpha)[-\ln(1-\alpha)]^{1/3}$	$[-\ln(1-\alpha)]^{1/3}$
Nucleation models-4-dimension	A4	$4(1-\alpha)[-\ln(1-\alpha)]^{1/4}$	$[-\ln(1-\alpha)]^{1/4}$
Geometrical contraction models-1-dimension	R1	1	α
Geometrical contraction models - sphere	R2	$2(1-\alpha)^{1/2}$	1-$(1-\alpha)^{1/2}$
Geometrical contraction models - cylinder	R3	$3(1-\alpha)^{1/3}$	1-$(1-\alpha)^{1/3}$
Nucleation models-2-Power law	P2	$2\alpha^{1/2}$	$\alpha^{1/2}$
Nucleation models-3-Power law	P3	$3\alpha^{2/3}$	$\alpha^{1/3}$
Nucleation models-4-Power law	P4	$4\alpha^{3/4}$	$\alpha^{1/4}$

Adapted from Criado et al. [21] and ICTAC Kinetics Committee Recommendations [18].

polymers MDPI

Table S3. Apparent activation energies, pre-exponential factors, and coefficients of determination from Coats–Redfern screening of Stage 2 (oxidative devolatilization) and Stage 3 (char oxidation) at four heating rates (20, 40, 60, and 80 K min⁻¹). Stage 1 (moisture) was not fitted.

**Stage 2 — oxidative devolatilization**	Code	20			40			60			80		
		E_a (kJ/mol)	Ln(A_0)	R^2	E_a (kJ/mol)	Ln(A_0)	R^2	E_a (kJ/mol)	Ln(A_0)	R^2	E_a (kJ/mol)	Ln(A_0)	R^2
Reaction order models-First order	F1	71.9	10.9	0.9985	78.5	10.8	0.9984	78.8	11.5	0.9981	82.5	11.4	0.9974
Reaction order models-Second order	F2	101.4	5.0	0.9996	108.6	4.9	0.9995	111.6	5.2	0.9995	114.5	5.3	0.9992
Reaction order models-Third order	F3	136.5	-2.2	0.9996	144.2	-2.1	0.9996	150.7	-2.3	0.9997	152.3	-1.9	0.9993
Diffusion models-One dimension	D1	106.4	5.4	0.9960	118.3	4.5	0.9959	115.7	5.9	0.9952	124.0	5.0	0.9933
Diffusion models-Two dimension	D2	120.3	3.2	0.9971	132.8	2.3	0.9970	131.1	3.6	0.9965	139.3	2.8	0.9951
Diffusion models-Three dimension	D3	136.7	1.4	0.9981	149.6	0.4	0.9980	149.2	1.5	0.9976	157.1	0.8	0.9966
Diffusion models-Four dimension	D4	125.7	3.6	0.9975	138.4	2.7	0.9974	137.1	3.9	0.9969	145.2	3.1	0.9957
Nucleation models-Two dimension	A2	31.0	18.0	0.9980	34.1	18.4	0.9978	34.2	19.0	0.9974	36.0	19.1	0.9964
Nucleation models-Three-dimension	A3	17.3	20.1	0.9970	19.4	20.7	0.9969	19.3	21.2	0.9963	20.5	21.4	0.9950
Nucleation models Fourth dimension	A4	10.5	20.9	0.9953	12.0	21.6	0.9954	11.9	22.1	0.9943	12.7	22.3	0.9925
Geometrical contraction models-One dimension phase boundary	R1	48.2	15.5	0.9950	54.0	15.5	0.9951	52.6	16.4	0.9942	56.7	16.2	0.9919
Geometrical contraction models -Contracting sphere	R2	59.3	14.1	0.9972	65.6	14.0	0.9971	64.9	14.8	0.9966	68.9	14.6	0.9953
Geometrical contraction models- Contracting cylinder	R3	63.3	13.7	0.9977	69.7	13.6	0.9976	69.4	14.4	0.9972	73.2	14.2	0.9961
Nucleation models-Power law	P2	19.1	20.1	0.9919	21.9	20.5	0.9924	21.1	21.2	0.9908	23.1	21.3	0.9876
Nucleation models-Power law	P3	9.4	21.2	0.9849	11.2	21.8	0.9868	10.6	22.4	0.9834	11.8	22.6	0.9788
Nucleation models-Power law	P4	4.6	21.4	0.9639	5.8	22.1	0.9724	5.3	22.6	0.9630	6.2	22.9	0.9570

**Stage 3 — char oxidation**	Code	20			40			60			80		
		E_a (kJ/mol)	Ln(A_0)	R^2	E_a (kJ/mol)	Ln(A_0)	R^2	E_a (kJ/mol)	Ln(A_0)	R^2	E_a (kJ/mol)	Ln(A_0)	R^2
Reaction order models-First order	F1	93.6	8.8	0.9859	60.2	14.9	0.9964	48.4	18.0	0.9978	27.9	20.5	0.9985
Reaction order models-Second order	F2	242.9	-16.8	0.9785	140.3	1.2	0.9941	117.0	7.9	0.9953	64.7	14.3	0.9968
Reaction order models-Third order	F3	443.0	-51.8	0.9756	247.0	-17.6	0.9929	208.6	-6.1	0.9941	113.3	5.6	0.9959
Diffusion models-One dimension	D1	49.7	16.6	0.9899	41.7	18.6	0.9964	31.9	20.7	0.9998	22.5	22.1	0.9999
Diffusion models-Two dimension	D2	79.3	12.3	0.9917	62.0	15.9	0.9975	48.9	18.7	0.9999	33.4	21.0	0.9999
Diffusion models-Three dimension	D3	133.2	4.6	0.9902	94.5	11.8	0.9975	76.5	16.0	0.9991	49.4	19.7	0.9995
Diffusion models-Four dimension	D4	96.4	10.9	0.9915	72.5	15.6	0.9976	57.8	18.9	0.9997	38.6	21.6	0.9998
Nucleation models-Two dimension	A2	40.9	17.3	0.9818	24.2	20.4	0.9945	18.2	22.0	0.9963	8.0	22.8	0.9960
Nucleation models-Three-dimension	A3	23.4	19.8	0.9754	12.2	21.9	0.9906	8.1	22.8	0.9921	1.4	22.3	0.9510
Nucleation models Fourth dimension	A4	14.6	20.9	0.9651	6.2	22.2	0.9801	3.1	22.7	0.9715	-1.9	—	0.9861
Geometrical contraction models-One dimension phase boundary	R1	19.0	20.8	0.9828	15.0	22.0	0.9929	9.9	22.9	0.9995	5.4	23.3	0.9994
Geometrical contraction models -Contracting sphere	R2	47.5	17.1	0.9889	33.4	20.0	0.9968	25.5	21.8	0.9993	14.9	23.1	0.9995
Geometrical contraction models- Contracting cylinder	R3	60.7	15.4	0.9883	41.4	19.1	0.9968	32.3	21.3	0.9988	18.8	23.0	0.9992
Nucleation models-Power law	P2	3.6	21.6	0.8910	1.6	21.9	0.8603	-1.0	—	0.9842	-3.2	—	0.9991
Nucleation models-Power law	P3	-1.5	—	0.7657	-2.9	—	0.9775	-4.7	—	0.9995	-6.1	—	0.9998
Nucleation models-Power law	P4	-4.1	—	0.9764	-5.1	—	0.9958	-6.5	—	0.9998	-7.5	—	0.9999

Note: "—" means the parameter is undefined because of a negative regression intercept. Stage 3 E_a is not stable with heating rate and is not used for design. The working Stage 2 model is F2 (mean E_a = 109.0 kJ mol^{-1}).
